# Dissociation of LAG-3 inhibitory cluster from TCR microcluster by immune checkpoint blockade

**DOI:** 10.3389/fimmu.2024.1444424

**Published:** 2024-08-21

**Authors:** Akiko Hashimoto-Tane, Edward P. Bowman, Machie Sakuma, Natsumi Yoneda, Katsuyuki Yugi, Rene de Waal Malefyt, Takashi Saito

**Affiliations:** ^1^ Laboratory of Cell Signaling, RIKEN Center for Integrative Medical Sciences, Yokohama, Japan; ^2^ Department of Oncology, Merck & Co., Inc., Rahway, NJ, United States; ^3^ Laboratory of Integrated Cellular Systems, RIKEN Center for Integrative Medical Sciences, Yokohama, Japan; ^4^ Laboratory of Cell Signaling, Immunology Frontier of Immunology, Osaka University, Suita, Japan

**Keywords:** LAG-3, T cell activation, immune checkpoint blockade, TCR microcluster, PD-1, inhibitory co-receptor

## Abstract

Lymphocyte activation gene (Lag)-3 is an inhibitory co-receptor and target of immune checkpoint inhibitor (ICI) therapy for cancer. The dynamic behavior of Lag-3 was analyzed at the immune synapse upon T-cell activation to elucidate the Lag-3 inhibitory mechanism. Lag-3 formed clusters and co-localized with T-cell receptor microcluster (TCR-MC) upon T-cell activation similar to PD-1. Lag-3 blocking antibodies (Abs) inhibited the co-localization between Lag-3 and TCR-MC without inhibiting Lag-3 cluster formation. Lag-3 also inhibited MHC-II-independent stimulation and Lag-3 Ab, which did not block MHC-II binding could still block Lag-3’s inhibitory function, suggesting that the Lag-3 Ab blocks the Lag-3 inhibitory signal by dissociating the co-assembly of TCR-MC and Lag-3 clusters. Consistent with the combination benefit of PD-1 and Lag-3 Abs to augment T-cell responses, bispecific Lag-3/PD-1 antagonists effectively inhibited both cluster formation and co-localization of PD-1 and Lag-3 with TCR-MC. Therefore, Lag-3 inhibits T-cell activation at TCR-MC, and the target of Lag-3 ICI is to dissociate the co-localization of Lag-3 with TCR-MC.

## 1 Introduction

The treatment of cancer patients with antibodies (Abs) against the inhibitory co-receptors CTLA-4 and PD-1 (or PD-L1) has become an efficient immunotherapy for many types of tumors (1). Immune checkpoint inhibitor (ICI) therapy has become a pillar of cancer treatment along with surgery, radiation, and chemotherapy. The target of ICIs is T cell-mediated immunity; ICIs block the inhibitory function mediated by inhibitory co-receptors (such as CTLA-4 and PD-1) on previously activated antigen (Ag)-specific T cells to reduce the full development of T-cell exhaustion. Inhibitory co-receptors are upregulated in chronic viral infection and cancer environments due to persistent stimulation via T-cell receptor (TCR), resulting in T-cell exhaustion (2). ICI treatment induces additional proliferative bursts of the exhausted T-cell precursors to be more active against tumors (3).

Although ICI therapy was a historic development for tumor therapy, there remain problems. First, the treatment is accompanied by immune-related adverse events. Potently activated T cells that enhance immune responses can increase susceptibility to autoimmune diseases such as diabetes (4). Second, the benefit of ICIs is still limited to a subset of patients (5), and a considerable proportion of patients showed unresponsiveness due to resistance mechanisms such as loss of neo-antigen, reduced MHC class I (MHC-I) expression, few infiltrated T cells, lower expression level of PD ligands, and immunosuppressive tumor microenvironment. Recent efforts have focused on combination treatments of different ICIs to overcome these difficulties (6, 7).

Other inhibitory co-receptors such as lymphocyte activation gene-3 (Lag-3), T-cell immunoglobulin and mucin domain-containing protein 3 (TIM-3), and T-cell immunoreceptor with Ig and ITIM domains (Tigit) have been focused on as next-generation ICIs (8). Lag-3 has a structure closely related to CD4 consisting of four Ig domains and binds to MHC class II (MHC-II) with a higher affinity than CD4 (9, 10). Recently, fibrinogen-like protein-1 (FGL1) was reported to bind to Lag-3 independently from MHC-II (11). *In vivo* treatment of tumor-bearing mice with MHC-II-blocking anti-Lag-3 (aLag-3) Ab resulted in reduced tumor size similar to anti-PD-1 (aPD-1) Ab (12). Since both Lag-3 and PD-1 are co-expressed on tumor-infiltrated T cells (12), the cocktail of aLag-3 and aPD-1 functioned to reduce tumor size more effectively than each treatment alone in preclinical model systems (13).

The first successful combinatorial ICI trial was a cocktail of aLag-3 and aPD-1 Abs. OPDUALAG^®^, the combination of relatlimab (anti-Lag3) and nivolumab (anti-PD-1), was recently approved for the treatment of adult and pediatric patients with unresectable or metastatic melanoma (13). The combination of favezelimab (anti-Lag3) and pembrolizumab (anti-PD-1) is in PhIII studies for colorectal cancer and non-Hodgkin’s lymphoma; the combination of fianlimab (anti-Lag3) and cemiplimab (anti-PD-1) is in PhIII studies for non-small cell lung cancer and melanoma. Bispecific Lag-3/PD-1 modalities have also progressed into clinical development (14). The most advanced Lag-3/PD-1 bispecific candidate is tebotelimab, which is a bispecific, tetravalent DART^®^ molecule that is in PhII/III studies. Although clinical trials support the combination benefit of a cocktail of aLag-3 and aPD-1 Abs or a bispecific modality bearing both specificities (15), the mechanism to augment tumor immunity is still unknown.

T cells form TCR microcluster (TCR-MC) upon recognition of antigen at the immunological synapse (IS) (16). TCR-MC consists of hundreds of TCRs at the interface between T cells and antigen-presenting cells (APCs) and induces T-cell activation by recruiting signaling molecules such as ZAP-70 kinases, LAT adaptors, and PLCγ effector molecules to the IS and induces the phosphorylation of these signaling molecules to initiate T-cell activation. Previously, we have shown that PD-1 forms cluster upon the interaction with PD-L1 expressed on APC. PD-1 clusters were co-localized with TCR-MCs and induced negative regulation by recruiting SHP-2 phosphatase (17). The co-localization between PD-1 clusters and TCR-MC was necessary and critical for the inhibitory function of PD-1 (17).

In this study, we performed an imaging analysis of the dynamic feature of Lag-3 at the IS upon T-cell activation to elucidate the mechanism of Lag-3-mediated inhibition of T-cell activation. We found that Lag-3 formed clusters that co-localized with TCR-MC similar to PD-1 and that the co-localization between the TCR-MC and Lag-3 was critical for Lag-3’s inhibitory function. We also analyzed the requirement for MHC-II and Lag-3’s structural requirements to mediate the inhibitory function. Furthermore, the inhibitory function of Lag-3 and PD-1 was analyzed using blocking aLag-3 Abs and bispecific Lag-3/PD-1 modalities to explore their potential application as a combinatorial ICI.

## 2 Materials and methods

### 2.1 Mice

Moth cytochrome *c* (MCC)-specific AND-TCR transgenic (tg) mice were provided by Dr. Stephen M. Hedrick (University of California, San Diego, CA, USA) courtesy of Dr. Ronald N. Germain (National Institutes of Health, MD, USA). Ovalbumin (OVA)-specific OT-I TCR tg mice were provided by Dr. William R. Heath (The Walter and Eliza Hall Institute of Medical Research, Melbourne, VIC, Australia). Lag-3-deficient (KO) mice were generated by the CRISPR/Cas9 procedure as shown in **Supplementary Figure 6**. The constitutive Lag-3 KO mice were generated using CRISPR/Cas9-mediated gene editing at Taconic Biosciences Inc. for the Research Laboratories of Merck & Co., Inc., Rahway, NJ, USA. Out of a panel of potential sgRNAs, two sgRNAs were selected with the lowest number of predicted off-target mutations (**Supplementary Figure 6**). The Cas9 mRNA, the proximal sgRNA, and the distal sgRNA were co-injected into C57BL/6 zygotes by pronuclear injection. Multiple founders were generated with deletions varying from 11 to 66 bp at the junction sites confirmed by DNA sequencing. The founder with the smallest deletion of 11 bp was chosen to establish a colony of wild-type, heterozygous, and homozygous Lag-3 KO mice by further breeding. Deletion of exon 3 by CRISPR/Cas9-mediated gene editing should result in the loss of function of the Lag-3 gene by deleting part of the extracellular domain and by generating a frameshift from exons 2 to exons 4 to 8, resulting in a premature stop codon in exon 4. The mice were kindly provided by Merck & Co., Inc., Rahway, NJ, USA. AND-TCR tg/Lag-3 KO mice were generated by crossing AND-TCR tg mice with Lag-3 KO mice for the analysis of Ag-specific responses.

C57BL/6 and B10.BR mice were purchased from CLEA Japan, Inc. (Tokyo, Japan) and Japan SLC Inc. (Shizuoka, Japan), respectively. All the mice were maintained in our animal facility under specific pathogen-free (SPF) conditions and treated in accordance with the ethical guidance of RIKEN Yokohama Institute.

### 2.2 Reagents and Abs

Anti-mouse aPD-1 monoclonal antibody (mAb) DX400 was provided by Merck & Co., Inc., Rahway, NJ, USA. Abs specific for anti-phospho-Akt (S473) (#9271), anti-phospho-Akt (T308) (#4056), anti-phospho-Erk1/2 (#9101), anti-Erk1/2 (#9102), anti-phospho-PLCγ1 (Y783) (#2821), and anti-β-actin (#4967) were obtained from Cell Signaling Technology (Danvers, MA, USA). Anti-phospho-LAT (Y191) (07–278) was from Merck Millipore (Darmstadt, Germany), anti-CD3ε (145–2C11) and anti-TCRβ (H57–597) were from BD Biosciences (San Jose, CA, USA), and anti-CD28 (PV-1) was from Abcam (Cambridge, UK).

aLag-3 mAbs (rat antibodies 28G10, C9B7W, 17D8, 9B7, and 1F3) were provided by Merck & Co., Inc., Rahway, NJ, USA. Rats were immunized with mouse Lag-3-mouse IgG2a (mIgG2a) protein (Enzo Life Sciences, Farmingdale, NY, USA; ALX-522–099) in adjuvant and sequentially boosted with mouse Lag-3 protein or mouse Lag-3-expressing Ba/F3 cells. Standard hybridoma techniques were used to generate a hybridoma panel; hybridomas were screened using cell-based binding ELISA to mLag-3-expressing Chinese hamster ovary (CHO) cells. Positive hybridomas were evaluated for their ability to block the interaction of mouse Lag-3-mIgG2a binding to MHC-II on the A20 mouse cell line.

Bispecific monovalent PD-1/Lag-3 Ab and PD-1/Lag-3 VHH were provided by Merck & Co., Inc., Rahway, NJ, USA. Complementary mutations in the CH3 domain of the respective mouse IgG1 backbones were used to form the bispecific heterodimer of the murinized anti-mouse PD-1 antibody MuDX400 (mIgG1) and the rat/mouse chimeric anti-mouse Lag-3 antibody 28G10 (mIgG1). Both backbone sequences further incorporated the D265A mutation into the mouse IgG1 backbone to minimize FcγR engagement. Three plasmids containing 1) the anti-PD-1 DX400 scFv (VL-VH) fused to mouse IgG1 Fc, 2) the rat anti-Lag-3 28G10 Fab fused to mouse IgG1 Fc, and 3) the rat anti-Lag-3 28G10 kappa light chain were co-transfected into HEK293 Expi cells, bispecific antibody purified via protein A chromatography followed by further polishing steps [e.g., preparative size-exclusion chromatography (SEC)] to achieve >95% purity by analytical SEC chromatography. The purity and identity of the molecule were confirmed by intact mass and capillary electrophoresis–sodium dodecyl sulfate (CE-SDS). Endotoxin levels were <2 EU/mg, and affinity to respective targets was confirmed by surface plasmon resonance (SPR).

Bispecific monovalent PD-1/Lag-3 VHH was provided by Merck & Co., Inc., Rahway, NJ, USA. The anti-mouse PD-1 VHH module was generated by immunizing a llama with recombinant mouse PD-1-His protein. A phage display library was constructed from RNA isolated from the peripheral blood mononuclear cells (PBMCs) of the immunized llama; the library was subjected to selection using biotinylated mouse PD-1-His. The resulting mouse PD-1 VHH panel was screened to identify an anti-PD-1 VHH that binds to mouse PD-1 on engineered and primary cells, blocks the interaction of mouse PD-1 with its ligands mouse PD-L1 and mouse PD-L2, lacks reactivity to related PD-1 structural family members, and shows functional bioactivity in a 3A9 cell-based mouse PD-1 bioassay.

The anti-mouse Lag-3 VHH module was generated by immunizing an alpaca with recombinant mouse Lag-3-His protein. A phage display library was constructed from RNA isolated from the PBMCs of the immunized alpaca. The phage display library was subject to selection using biotinylated mouse Lag-3-His. The resulting mouse Lag-3 VHH panel was screened to identify an anti-mouse Lag-3 VHH that binds to mouse Lag-3, blocks the interaction of mouse Lag-3 with MHC class II, lacks reactivity to related Lag-3 structural family members, and shows functional bioactivity in a Lag-3 expressing 3A9 cell-based bioassay.

The selected anti-mouse PD-1 and anti-mouse Lag-3 modules were formatted using a 35 amino acid Gly-Ser linker ([GGGGS]x7) into a bispecific monovalent anti-mouse PD-1/anti-mouse Lag-3 construct. A third anti-mouse albumin module to enable half-life extension when dosed *in vivo* was attached with a second 35 amino acid Gly-Ser linker to construct the final trivalent species used in this manuscript. The order of VHH modules and linkers from the N-terminus to the C-terminus was PD-1:GS linker:Lag-3:GS linker:Albumin.

### 2.3 Construction and transduction

All the expression vectors were prepared by PCR subcloning. The retrovirus vector pMXs (provided by Dr. Toshio Kitamura, Tokyo University) was used for the expression of fluorescent-labeled molecules. Lag-3 and PD-1 were cloned from the cDNA of activated CD4 T cells. Green fluorescent protein (GFP) and cyan fluorescent protein (CFP) were derived from pEGFP-N1 or pECFP-N1 (BD Clontech, Mountain View, CA, USA) and HaloTag from HaloTag pHT2 vector (Promega, Madison, WI, USA), respectively. Lag-3 deletion mutants were as follows: Lag3(ΔCP) consisted of 1–474aa, Lag-3(234) consisted of 168–506aa, Lag-3(1) consisted of 1–167aa, and Lag-3(1)hCD22(345) contained Lag-3’s 1–167aa, human CD22’s 3rd, 4th and 5th Ig domains, and Lag-3’s 427–506aa successively (as illustrated in **Supplementary Figure 4A**).

### 2.4 Cell preparation and stimulation

T cells from the spleen and lymph node of AND-TCR tg or OT-I TCR tg mice were purified using Mojo Sort Isolation Kits (BioLegend, San Diego, CA, USA). To prepare purified naïve T cells, the cells were stained with fluorescent-labeled Abs and sorted by BD FACS Aria III (Becton, Dickinson and Company, Franklin Lakes, NJ, USA). Naïve T cells were negatively gated by B220, NK1.1, TCRγδ, CD49, CD11c, CD11b, Ter-119, CD25, and CD45RO and positively sorted by CD62L^high^, CD44^low^, and CD4/CD8. The purified T cells were stimulated with plate-bound anti-CD3ε (2C11) (10 μg/mL) and anti-CD28 (PV-1) Abs (5 μg/mL) in RPMI-1640 (Sigma, Darmstadt, Germany) with 10% fetal bovine serum (FBS) and 1% Penicillin-Streptomycin (Invitrogen, Carlsbad, CA, USA). AND TCR tg T cells were similarly purified and cultured with irradiated spleen cells from B10.BR mice and 3 μM MCC (88–103) (ANERADLIAYLKQATK, Cambridge Bioscience, Cambridge, UK) or a low-affinity mutant peptide [MCC(K99A)]. OT-I TCR tg T cells were similarly stimulated with OVA peptide (SIINFEKL) and irradiated C57BL/6 spleen cells. Bone marrow dendritic cells (BMDCs) were prepared by culturing bone marrow (BM) cells in 10 ng/mL granulocyte-macrophage colony-stimulating factor (GM-CSF) (PeproTech, Cranbury, NJ, USA) for 10 days. One day prior to the experiment, BMDCs were harvested, and 1 μg/mL lipopolysaccharide (LPS) (Sigma) was added to mature the BMDCs; thereafter, Ag peptides were added to BMDCs to use them as APC. The 2D12 T-cell hybridoma was derived from AND-TCR tg T cells and was used for functional analysis. 2D12 T-cell hybridoma was stimulated with MCC peptide and DC line DC-1 similar to AND-TCR tg T cells.

### 2.5 ELISA

Purified T cells (5 × 10^4^ cells/well) were stimulated with plate-bound anti-CD3ε and anti-CD28 Abs with or without blocking Abs for 48 hours. AND-TCR tg T cells were stimulated with irradiated B10BR splenocytes (5 × 10^5^ cells/well) and MCC peptide, whereas OT-I TCR tg T cells were stimulated by irradiated C57BL/6 splenocyte and OVA peptide under a similar condition. The amount of IL-2 in the culture supernatant was analyzed by ELISA (BD Biosciences).

### 2.6 Planar bilayer system

Lag-3-GFP, Lag3(ΔCP)-GFP, Lag-3(234)-GFP, Lag-3(1)-GFP, Lag-3(1)hCD22(345)-GFP, or Lag-3-GFP and PD-1-CFP were transduced into AND-TCR tg/Lag-3 KO T cells, which were fluorescence-activated cell sorting (FACS)-purified and stimulated with anti-CD3ε/CD28 mAbs for 2 days using a retrovirus-mediated gene transfer system. The infected cells were sorted for GFP^+^ cells using a FACS Aria on day 5 after infection and stained with a non-blocking anti-TCRβ Ab (H57–597) labeled with Dylight549 for live imaging. The preparation of a planer bilayer containing glycosylphosphatidylinositol (GPI)-anchored mouse MHC-II I-Ek and ICAM-1 was described previously (18). The Olympus (Tokyo, Japan) total internal reflection fluorescence microscopy (TIRF) system was configured on an IX81 microscope. The following were used: TIRF objective lens (UAPON ×100, NA1.49, oil, Olympus), triple excitation lasers; DPSS 445-nm, 488-nm, and 561-nm laser system (Melles Griot, Rochester, NY, USA; 85BCD020 and 85YCA020); and CCD camera (ORCA flash4.0, Hamamatsu Photonics, Shizuoka, Japan). Triple color videos were taken using MetaMorph software (Molecular Devices, San Jose, CA, USA).

### 2.7 Image analysis

Fiji (freeware), IMARIS (BitPlane), and Excel (Microsoft) were used for statistical analysis of fluorescent clusters. All the movies taken using MetaMorph were normalized in Fiji, as follows: subtracted background using rolling background subtraction method, automatically adjusted contrast at maximum expansion timepoint, converted to 8 bits, and saved as sequential separated images. The sequential images were analyzed on IMARIS. First, co-localized areas were detected by an additional module “coloc.”, which extracted co-localized areas based on pixel fluorescence intensity histograms. Lag3-GFP clusters, TCR-MC, and co-localized images were detected by evaluating cluster intensity and shape. The time course of cluster number and intensities were exported as csv files. All images were quantified from more than 50 individual cells. Finally, the data in the csv files were arranged by Python programs written by Katsuyuki Yugi and summarized in Excel.

### 2.8 Western blotting

2D12 T-cell hybridoma was cultured in RPMI-1640 (Sigma) with 10% FBS and 1% Penicillin-Streptomycin (Invitrogen, Carlsbad, CA, USA). Lag-3-IRES-hCD8 and PD-1-IRES-GFP were transfected into 2D12 T-cell hybridomas by a retrovirus gene transfer system. The transfected 2D12 cells were stimulated with MCC (5 μM) pulsed DC line DC-1 expressing MHC-II I-Ek and ICAM-1 for 5 min or 15 min and lysed. The cell lysates were boiled in a sample buffer and subjected to SDS–polyacrylamide gel electrophoresis (SDS-PAGE). The gels were developed for Western blotting with Abs described in the Reagents and Abs section.

### 2.9 Confocal microscopy analysis

The distribution of Lag-3-GFP in cell–cell interaction conditions was analyzed by confocal microscopy. Images of the interaction between T cells and activated B cells, which had been stimulated with LPS for 24 hours, were taken using a Leica TCS SP5 microscope with an oil immersion objective (HCX PL APO 100×, NA1.44, oil, Leica, Wetzlar, Germany).

### 2.10 Flow cytometry analysis

Purified naïve AND-TCR tg T cells were stimulated by irradiated B10BR splenocyte and MCC peptide for 9 days. On days 0, 1, 2, 3, 7, and 9, the cells were stained by anti-mouse Lag-3 (clone 28G10), anti-mouse PD-1 (clone DX400), anti-mouse TIGIT (clone 1G9), anti-mouse CD3e (clone 2C11), and anti-mouse CD25 (clone MBSA43). The stained cells were analyzed on BD LSR Fortessa X-20 (Becton, Dickinson and Company).

### 2.11 Statistical analysis

Statistical significance was determined by a two-tailed unpaired Student’s *t*-test using KaleidaGraph (Synergy Software). Data are presented as the mean ± SEM values. p < 0.05 was considered statistically significant.

## 3 Results

### 3.1 Lag3 cluster accumulation in TCR microclusters upon T-cell stimulation

**Figure 1:**
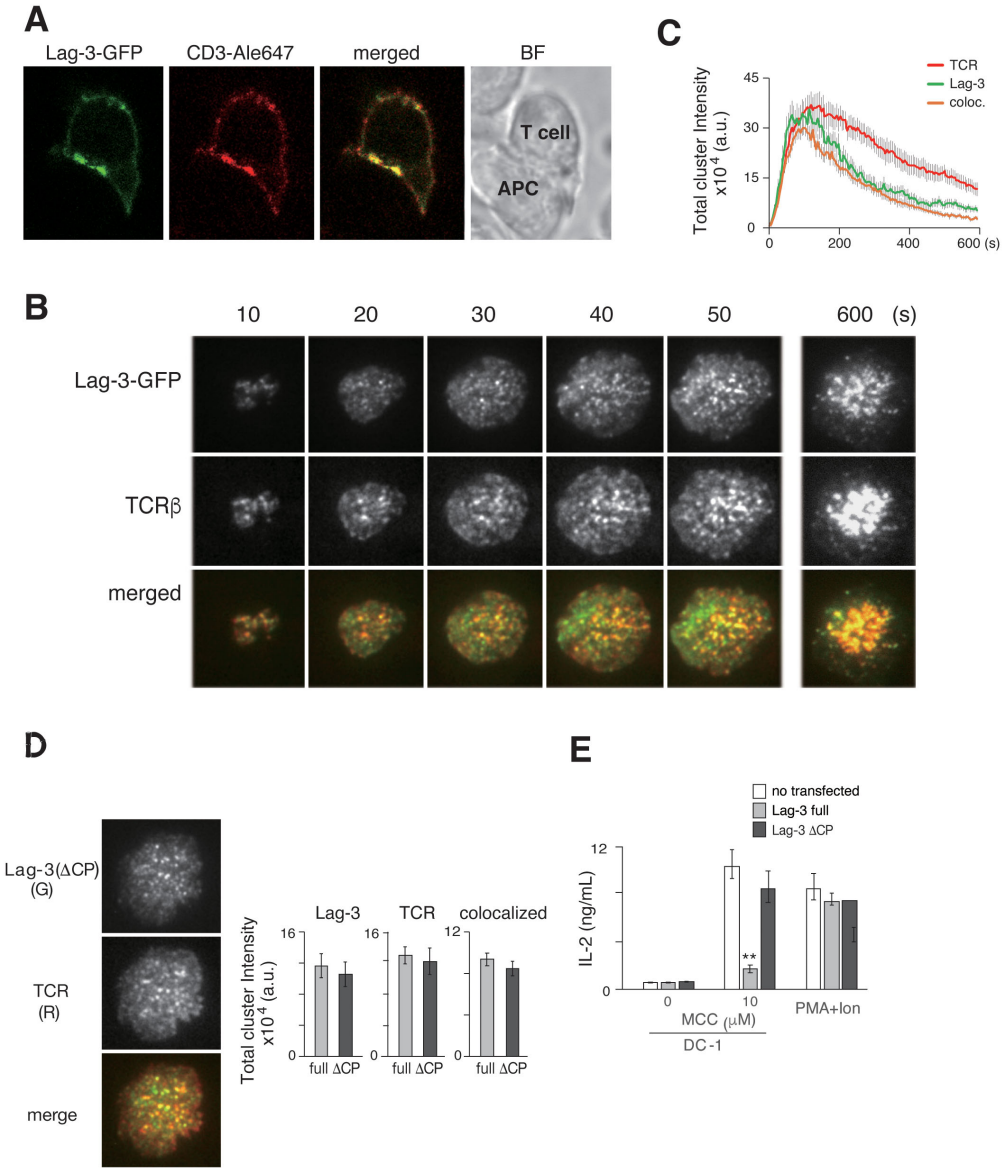
Lag-3 forms clusters co-localized with TCR-MCs. **(A)** T cells from AND-tg/Lag-3 KO mice expressing Lag-3-GFP were incubated with MCC(K99A) peptide-loaded APC for 10 min and fixed. The images from side of conjugated cells were taken by confocal microscopy. Representative images of Lag-3-GFP (green), TCR stained by aCD3ε Ab (red), and merged, and the bright field (BF) revealed Lag-3 accumulation at immune synapse. **(B)** Lag-3-GFP-expressing AND-tg T cells were analyzed on planer bilayer, and the TIRF images every 10 sec (s) are shown. Lag-3-GFP formed clusters and co-localized with TCR-MCs (TCRβ). Single-colored clusters in panels **(B, D, F)** are expressed in gray for better resolution. **(C)** The total cluster intensity, a metric derived from cluster number and each cluster’s intensity, is plotted as a function of time for the images in panel **(B)** Lag-3-GFP is shown in green and TCRβ in red, and yellow denotes overlapping co-association. **(D)** 2D12 T-cell hybridoma cells expressing Lag3(ΔCP)-GFP were stained with H57 for TCR. Left: the images of Lag-3(ΔCP)-GFP (green) and TCR (red), merged on planer bilayer, are shown. Lag-3(ΔCP)-GFP showed intact cluster formation and co-localization with TCR-MCs. Right: statistical analysis of the peak total cluster intensity values, Lag-3-GFP, TCR, and co-localized clusters for Lag-3-GFP (gray) and Lag3(ΔCP)-GFP (black) are plotted. **(E)** IL-2 production from Lag-3-GFP or Lag-3(ΔCP)-GFP expressing 2D12 hybridoma cells. The cells were stimulated with 10 μM MCC peptide-pulsed DC-1 cells or PMA plus ionophore for 24 hours. Experiments were performed in triplicate, and the average ± SD is shown. **p < 0.01. Single-colored clusters in panels **(B, D)** as well as **Figures 2A, D**, **3A**, **4A**, **6B**. **Supplementary Figure 4A** is expressed in gray for better resolution. TCR-MCs, T-cell receptor microclusters; APC, antigen-presenting cell; TIRF, total internal reflection fluorescence microscopy; MCC, moth cytochrome *c*; PMA, phorbol myristate acetate; Ab, antibody.

It is known that Lag-3 expression in T cells is upregulated upon TCR stimulation. Lag-3 was expressed on the cell surface of AND TCR tg T cells upon stimulation with MCC peptide and APC in a similar time course to the early activation marker CD25 (**Supplementary Figure 1**). Based on the kinetics of the expression, imaging analysis of Lag-3 was performed 5–7 days after antigen stimulation. T cells were purified from AND-tg/Lag-3 KO mice and stimulated with anti-CD3/CD28 mAbs. T cells were then retrovirally transfected with Lag3-GFP, re-activated with MCC-pulsed APC, sorted for GFP^+^ cells by FACS, and analyzed by confocal microscopy. The expression of Lag-3 on the transfectants was similar to the level of endogenous Lag-3 on activated T cells from wild-type mice (**Supplementary Figure 1**). The images clearly demonstrated that Lag-3 accumulated at the interface between a T cell and an APC, suggesting that Lag-3 functions on TCR-mediated signaling at the immune synapse (**Figure 1A**). Simultaneous analysis of Lag-3-GFP and TCRs was performed on a planer bilayer membrane containing peptide-MHC-II (pMHC) and ICAM-1, which mimics the surface of an APC. The live images were taken by total internal reflection fluorescence microscopy (TIRFM). From the very earliest time points, Lag-3 generated clusters that overlapped with TCR-MCs. The Lag-3 cluster number increased along with cell expansion on the planar membrane upon activation, eventually moving to the center of the interface, which generated the central supramolecular activation cluster (cSMAC). During the process, most of the Lag-3-GFP clusters remained co-localized with TCR-MCs (**Figure 1B**).

The number and intensity of TCR-MC and Lag-3 clusters were evaluated using IMARIS software. The procedure for numerical analysis of imaging data is described in **Supplementary Figure 2**. Cluster number is not the most sensitive metric because of the large variation in cluster intensities. For example, very small and large clusters were equally counted as one. Therefore, the parameter of “total cluster intensity” (the cluster number value multiplied by the average cluster intensity) was used to show the magnitude of cluster formation and co-localization. The total cluster intensities of Lag-3 and TCR were plotted against time in **Figure 1C**. A simpler metric, the peak value of the “total cluster intensity” time course, will be plotted as a bar graph for the remainder of the manuscript.

### 3.2 Lag-3 cytoplasmic domain is critical for inhibitory function but not cluster formation

To analyze the structural requirement for Lag-3’s cluster formation and function, mutant Lag-3 without a cytoplasmic domain [Lag3(ΔCP)] was analyzed (**Figure 1D**). On the same planer bilayer, Lag-3(ΔCP) formed clusters almost identical to those of wild-type Lag-3. Lag-3 or Lag-3(ΔCP) was transfected into 2D12 T-cell hybridoma lacking Lag-3 expression and transfectants stimulated with MCC-pulsed APC to analyze structure–function relationship. Hybridoma T cells expressing full-length Lag-3 strongly reduced IL-2 production, whereas T cells expressing Lag-3(ΔCP) exhibited no inhibition of IL-2 production (**Figure 1E**) similar to previous reports (19). These data clearly indicate that the Lag-3 cluster formation depends on Lag-3’s extracellular domain and that Lag-3’s inhibitory function is mediated through its intracellular domain.

### 3.3 Quantitative analysis of Lag-3 clusters using aLag-3 Abs

**Figure 2:**
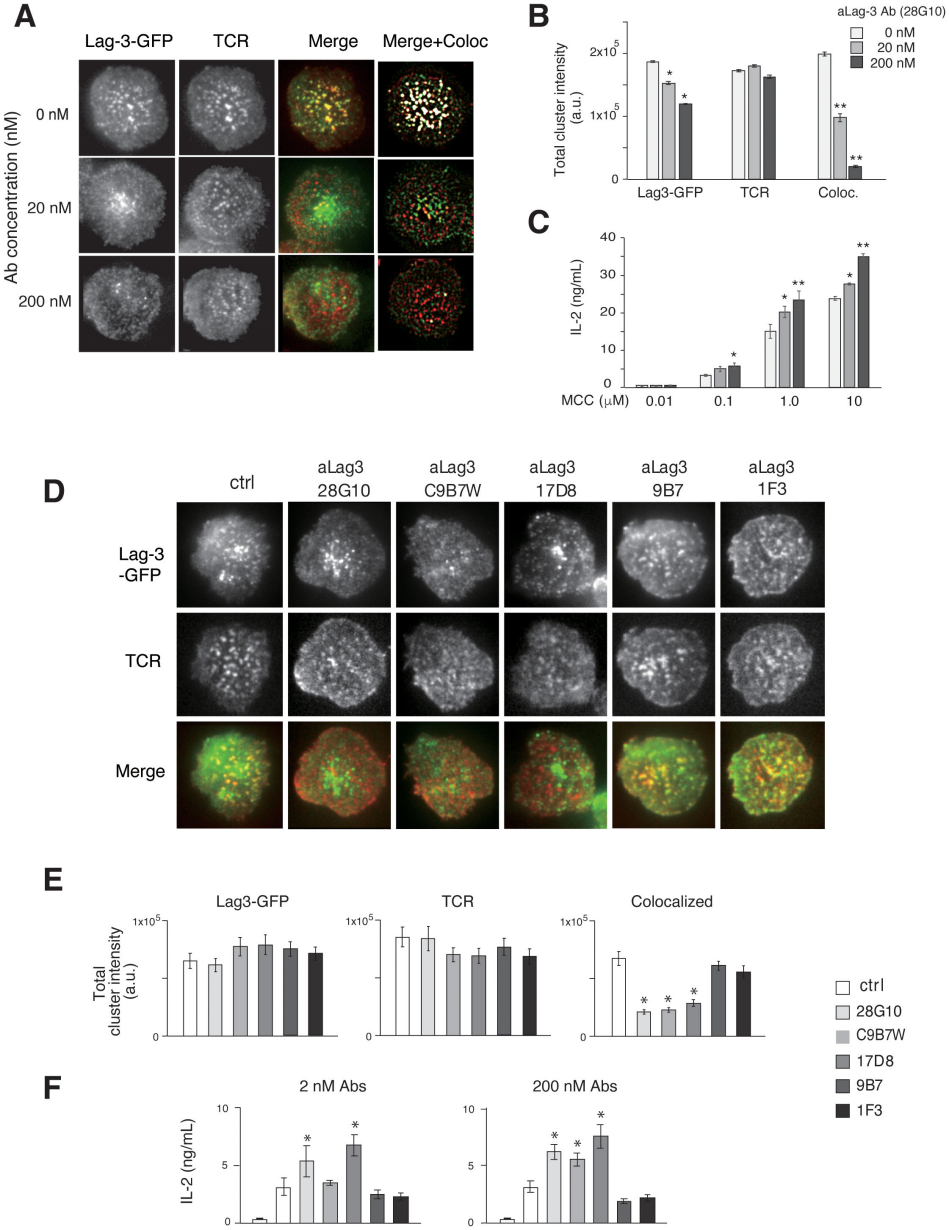
Effects of aLag-3 blocking Abs on Lag-3 cluster formation. **(A)** The Lag-3-GFP expressing AND-tg/Lag-3 KO T cells were pre-incubated with 20 nM or 200 nM aLag-3 Ab (28G10) and examined on planer bilayers. The representative images of Lag-3-GFP (green) and TCR (red), merged, and background subtracted merged with co-localized (with TCR-MC) image (white) at 60 sec after activation started are shown. **(B)** The peak value of total cluster intensity in T cells from panel **(A)** is plotted. **(C)** IL-2 production by naïve AND-tg T cells stimulated by graded concentrations (0.01 μM to 10 μM) of MCC and APC in the presence or absence of aLag-3 Ab at 48 hours are plotted. **(D–F)** Lag-3-GFP expressing AND-tg T cells were treated with a panel of aLag-3 Abs (28G10, C9B7W, 1F3, 9B7, and 17D8). **(D)** Representative images of Lag-3 cluster and TCR-MC formation after Abs were added at time 0, and the images were taken 60 sec after activation. **(E)** The peak value of total cluster intensities of panel **(D)** is plotted. **(F)** IL-2 production by T cells at 48 hours in the presence of aLag-3 Abs is shown. The leftmost columns represent unstimulated conditions. Experiments in panels **(C, F)** were performed in triplicate, and the average ± SD is shown. * and ** mean p < 0.1 and 0.01. aLag-3, anti-Lag-3; Abs, antibodies; TCR, T-cell receptor; TCR-MC, T-cell receptor microcluster; MCC, moth cytochrome *c*; APC, antigen-presenting cell.

To quantitatively analyze Lag-3 clusters and TCR-MCs, we analyzed the effect of adding aLag-3 mAb 28G10 (20), which strongly blocks the interaction between Lag-3 and MHCII, to the planer bilayer system. Whereas pre-treatment of Lag-3-expressing T cells with 28G10 resulted in slightly reduced Lag-3 cluster formation, the co-localization of Lag-3 clusters with TCR-MCs was strongly inhibited in a dose-dependent manner (**Figures 2A, B**). It is noteworthy that 28G10, which was selected for the inhibition of Lag-3/MHC-II association, also blocked the interaction between Lag-3 clusters and TCR-MC upon activation. Functionally, the addition of 28G10 induced the enhancement of IL-2 production in a dose-dependent manner (**Figure 2C**), indicating that the co-localization of Lag-3 with TCR-MCs is correlated with Lag-3-mediated inhibition of T-cell activation.

The effect of other Lag-3 mAbs with different attributes was examined (**Figures 2D–F**). 28G10 binds to the first Ig domain of Lag-3 and blocks Lag-3/MHC-II binding (IC50 = 2.3 μg/mL) (21). In contrast, C9B7W and 17D8 weakly/minimally block Lag-3/MHC-II interaction (**Supplementary Figure 3**); however, they both strongly inhibited co-localization between Lag-3 cluster and TCR-MCs (**Figures 2D, E**) and enhanced IL-2 production (**Figure 2F**). The aLag-3 Abs 9B7 and 1F3 weakly/minimally blocked Lag-3/MHC-II interaction (**Supplementary Figure 3**) and did not impact Lag-3 cluster co-localization with TCR-MCs nor IL-2 production (**Figures 2D–F**). These data indicate that inhibition of Lag-3 cluster co-localization with TCR-MC, but not blocking Lag-3/MHC-II interaction, is correlated with the functional upregulation of IL-2 production, suggesting that MHC-II binding by Lag-3 is not necessarily required for its inhibitory function.

### 3.4 Lag-3-mediated inhibitory function in an MHC-II-independent manner

**Figure 3:**
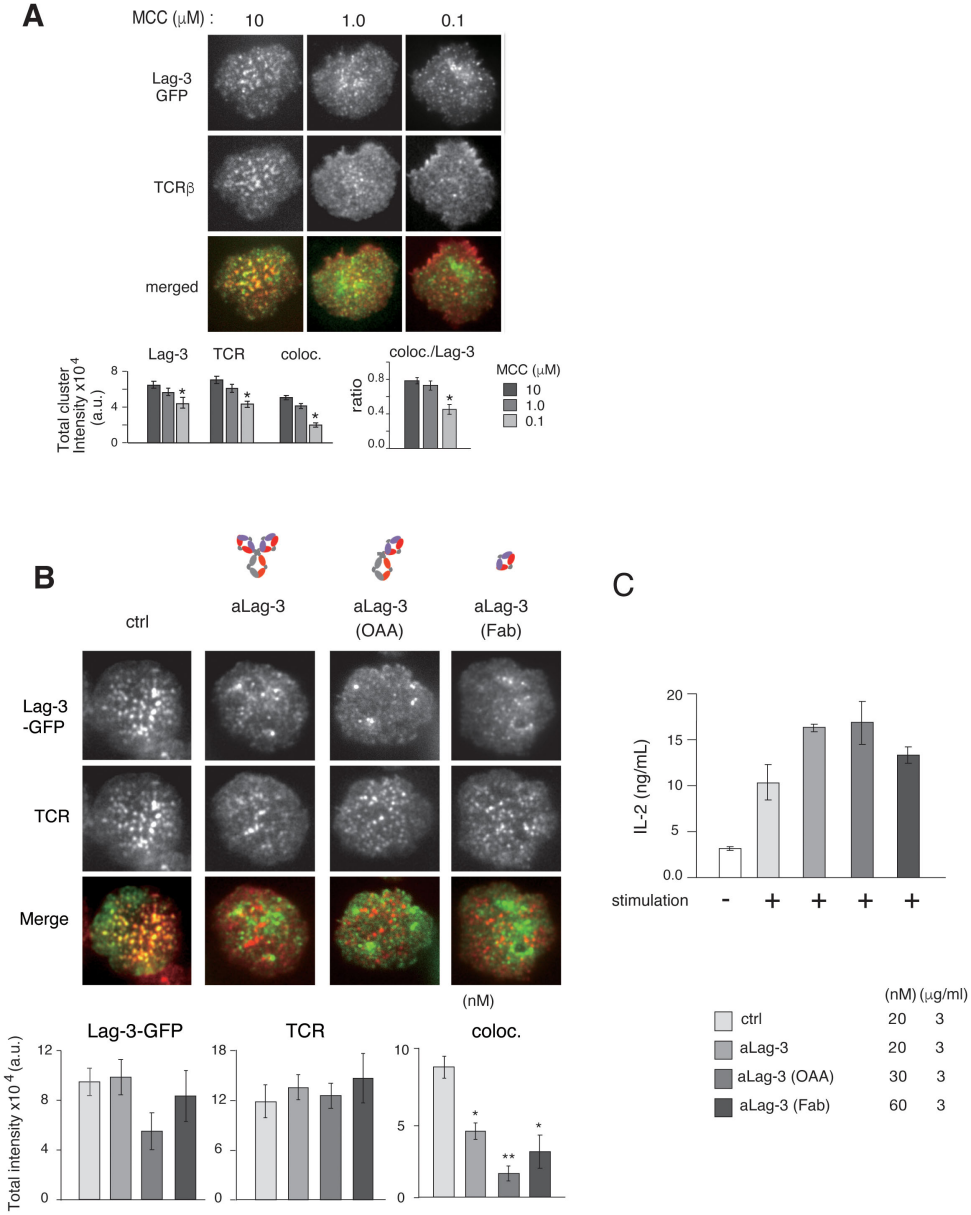
Inhibitory function of various forms of aLag-3 Ab on cluster formation. **(A)** Effects of Ag concentration. Top: the images of Lag-3-GFP (green) and TCR (red) of AND-tg/Lag-3 KO T cells on the planer bilayers loaded with constant amount of MHC-II (I-E^k^) (200 molecules/μm^2^) and increasing Ag peptide concentrations from 100 nM to 10 μM. Bottom: the results of statistical analysis. The “coloc./Lag-3” ratio was calculated by dividing co-localized cluster intensity by Lag-3-GFP intensity and indicates the efficiency of co-localization of Lag-3-GFP with TCR-MC depending on TCR stimulation strength. Experiments were performed in triplicate, and the average ± SD is shown. * and ** mean p < 0.1 and 0.01, respectively. **(B)** Lag-3-GFP expressing AND-tg T cells were pre-incubated with 20 nM for each control Ab or various aLag-3 Ab formats [i.e., conventional bivalent Ab, monovalent one-armed Ab (OAA), and monovalent Fab]. Images of cluster formation were taken on planer bilayer, and peak value of total cluster intensities is plotted. **(C)** IL-2 production by naïve AND-tg T cells stimulated by 10 μM MCC plus APC with or without various aLag-3 Ab formats at 48 hours is shown. aLag-3, anti-Lag-3; Ab, antibody; TCR, T-cell receptor; TCR-MC, T-cell receptor microcluster; MCC, moth cytochrome *c*; APC, antigen-presenting cell.

The synchronous movements of Lag-3 clusters and TCR-MCs could be mediated by sharing the same ligand MHC-II; however, we found that the efficiency of cluster co-localization depends on the dose of antigen peptide even in the presence of the same concentration of MHC-II on planer bilayer (**Figure 3A**). Both Lag-3 cluster formation and co-localization with TCR-MC were reduced when T cells were stimulated with low doses of antigen peptide, suggesting that Lag-3 cluster formation and co-localization with TCR-MCs may depend on activated TCRs rather than MHC-II at the immune synapse.

The importance of Lag-3 binding to MHC-II on Lag-3 cluster formation and inhibitory function was further investigated, first by analyzing Lag-3 mutants lacking MHC-II binding and second by analyzing Lag-3 function in MHC-I-restricted T-cell responses.

It has been shown that the first Ig domain of Lag-3 is the MHC-II-binding site (20). A mutant Lag-3 lacking the first Ig domain [Lag-3(234)], a mutant Lag-3 consisting of only the first Ig domain [Lag-3(1)], and a Lag-3 mutant with other Ig domains of human CD22 [Lag-3(1)hCD22(345)] to keep the similar molecular size as Lag-3 were constructed, and transfectants expressing these Lag-3 mutants were analyzed (**Supplementary Figures 4A, B**). Imaging analysis of these mutants on a planar bilayer revealed that all mutants similarly generated Lag-3 clusters, but the Lag-3 clusters failed to co-localize with TCR-MCs and also lost the ability to inhibit IL-2 production (**Supplementary Figures 4A, B**). The result of Lag-3(234) indicates that the first Ig domain is important for both co-localization of Lag-3 clusters with TCR-MCs and inhibitory function, while the results of Lag-3(1) and Lag-3(1)hCD22(345) indicated that the first Ig domain is not solely enough for both co-localization and inhibitory functions.

Next, MHC-I-restricted CD8 T-cell responses using OVA-specific OT-I tg T cells were investigated to evaluate whether Lag-3/MHC-II association is critical for inhibitory function (**Supplementary Figure 4C**). Previous analysis of Lag-3 on CD8 T cells suggested that the inhibition of T-cell activation is mediated by the intrinsic effect of Lag-3 (22); we confirmed the importance of MHC-II as the ligand for the function of Lag-3 on CD8 T cells. OT-I T cells were activated by wild-type (WT) and MHC-II KO APC in the presence or absence of 28D10, which inhibits Lag-3/MHC-II interaction. Both naïve and effector OT-1 T cells produced increased amounts of IL-2 upon stimulation with WT APCs by the addition of aLag-3 Ab, aPD-1 Ab, or the mixture of both Abs (**Supplementary Figure 4C**). Naïve T cells activated with MHC-II-KO APCs produced similar amounts of IL-2 to T cells activated with WT APC; however, the increased IL-2 production by the addition of aLag-3 was much smaller by T cells activated with MHC-II-KO APCs compared to WT APCs. In contrast, IL-2 production by effector T cells upon stimulation with MHC-II-KO APC was unchanged by the addition of aLag-3 Ab regardless of the presence of aPD-1. These results from effector T cells indicate that whereas the augmentation by aLag-3 on effector T cells was dependent on MHC-II-binding, the results on naïve T cells suggest the presence of MHC-II-independent inhibitory mechanism.

Supporting this idea, Lag-3 inhibitory effect on IL-2 production was also observed in the system of plate-bound anti-TCRβ Ab stimulation, which is completely free from MHC-II (**Supplementary Figure 4D**). T cells expressing Lag-3 and PD-1 reduced IL-2 production upon stimulation by plate-bound anti-TCRβ Ab. The inhibited response was recovered by the addition of aLag-3 Ab but not aPD-1 Ab. Therefore, these data indicate that Lag-3 inhibits TCR activation at least in part in an MHC-II-independent manner.

### 3.5 Relationship between Lag-3 and PD-1 on cluster formation and inhibitory function

Whereas aPD-1 Ab inhibited PD-1 cluster formation as previously shown (17), Lag-3 clusters remained after aLag-3 treatment (**Figure 3B**). To exclude the possibility that the Lag-3 clusters remaining after aLag-3 treatment were due to aggregation caused by the bivalency of the Ab, monovalent one-armed 28G10 Ab (OAA) and monovalent 28G10 Fab were examined using the planar bilayer system. Treatment of T cells with OAA or Fab did not reduce Lag-3 clusters per se; however, they inhibited co-localization of Lag-3 clusters with TCR-MCs (**Figure 3B**). Furthermore, these modalities enhanced T-cell activation as evidenced by increased IL-2 production (**Figure 3C**). Therefore, Lag-3 clusters remaining after aLag-3 treatment were not due to bivalent aLag3 Ab-mediated Lag-3 aggregation. These data clearly showed that OAA and Fab of aLag-3 Ab modalities are potent to both disrupt Lag-3 cluster/TCR-MC interactions and block Lag-3’s inhibitory function.

**Figure 4:**
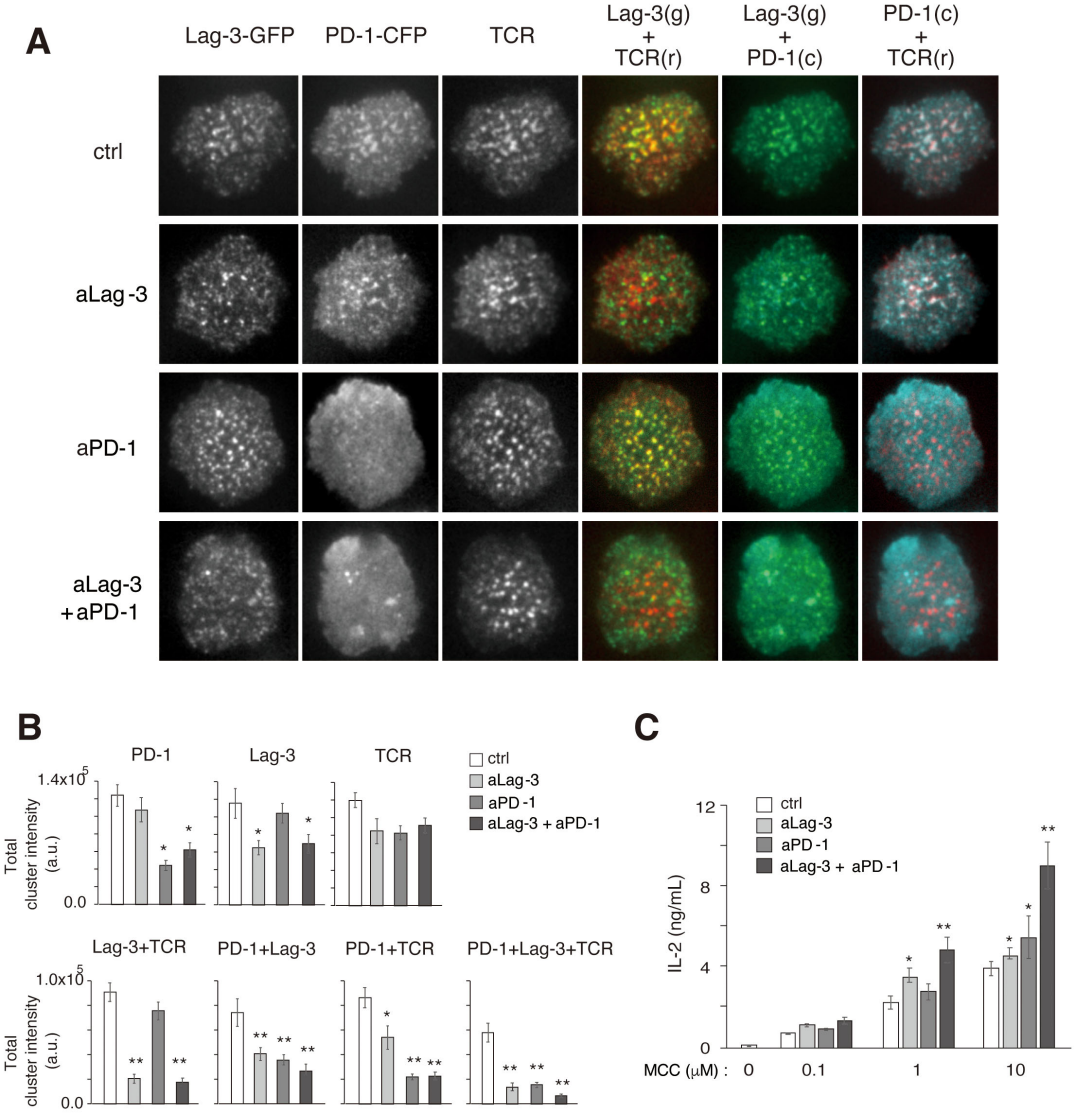
Simultaneous analysis of Lag-3, PD-1, and TCR cluster formation. **(A)** Representative images of Lag-3-GFP, PD-1-CFP, TCR, co-localization of Lag-3-GFP and TCR, co-localization of PD-1-CFP and TCR, and co-localization of PD-1-CFP and Lag-3-GFP following treatment with control IgG (ctrl), aLag-3 Ab (28G10), aPD-1 Ab (DX400), or the cocktail of aLag-3 Ab and aPD-1 Ab (20 nM) are shown. **(B)** The peak values of total cluster intensities of the images in panel **(A)** are plotted. **(C)** IL-2 production by naïve AND-tg T cells upon stimulation with MCC (0 μM, 0.1 μM, 1 μM, or 10 μM) and APC in the presence of 20 nM of control IgG (ctrl IgG), aLag-3, aPD-1, or the cocktail of aLag-3 and aPD-1 Abs (20 nM each) for 48 hours are shown. Experiments were performed in triplicate, and the average ± SD is shown. * and ** mean p < 0.1 and 0.01, respectively, using Student’s *t*-test. The cocktail showed combination-enhancing effects. TCR, T-cell receptor; MCC, moth cytochrome *c*; APC, antigen-presenting cell.

Several reports have shown that the cocktail of aPD-1 plus aLag-3 Abs shows a combination benefit of enhancing anti-tumor T-cell responses *in vivo* (23, 24). To elucidate the molecular mechanism, simultaneous imaging analysis of Lag-3, PD-1, and TCR was performed by triple-colored TIRFM, and the effect of blocking Abs was examined. AND-tg T cells co-expressing Lag-3-GFP and PD-1-CFP were stained by H57(Fab)-Dylight555 and analyzed on a planer bilayer containing PD-L1 and pMHC-II (**Figures 4A, B**). As expected, Lag-3, PD-1, and TCR-MC were all co-localized with each other upon Ag stimulation. Treatment with aLag-3 Ab (28G10) alone inhibited co-localization of Lag-3 clusters and TCR-MCs but had no significant effects on PD-1 clusters or TCR-MCs. Similarly, treatment with aPD-1 Ab alone inhibited PD-1 cluster formation but had no significant effects on Lag-3 clusters or TCR-MCs. Now, the cocktail of aLag-3 plus aPD-1 Abs showed additive effects of the respective individual treatment: inhibition of co-localization of Lag-3 and TCR-MCs and the inhibition of PD-1 cluster formation. However, no synergistic effects of the cocktail of aLag-3 and aPD-1 on cluster formation were detected by the imaging analysis over the expected additive effect (**Figure 4B**); IL-2 augmentation by the combination treatment of the Abs was observed (**Figure 4C**).

**Figure 5:**
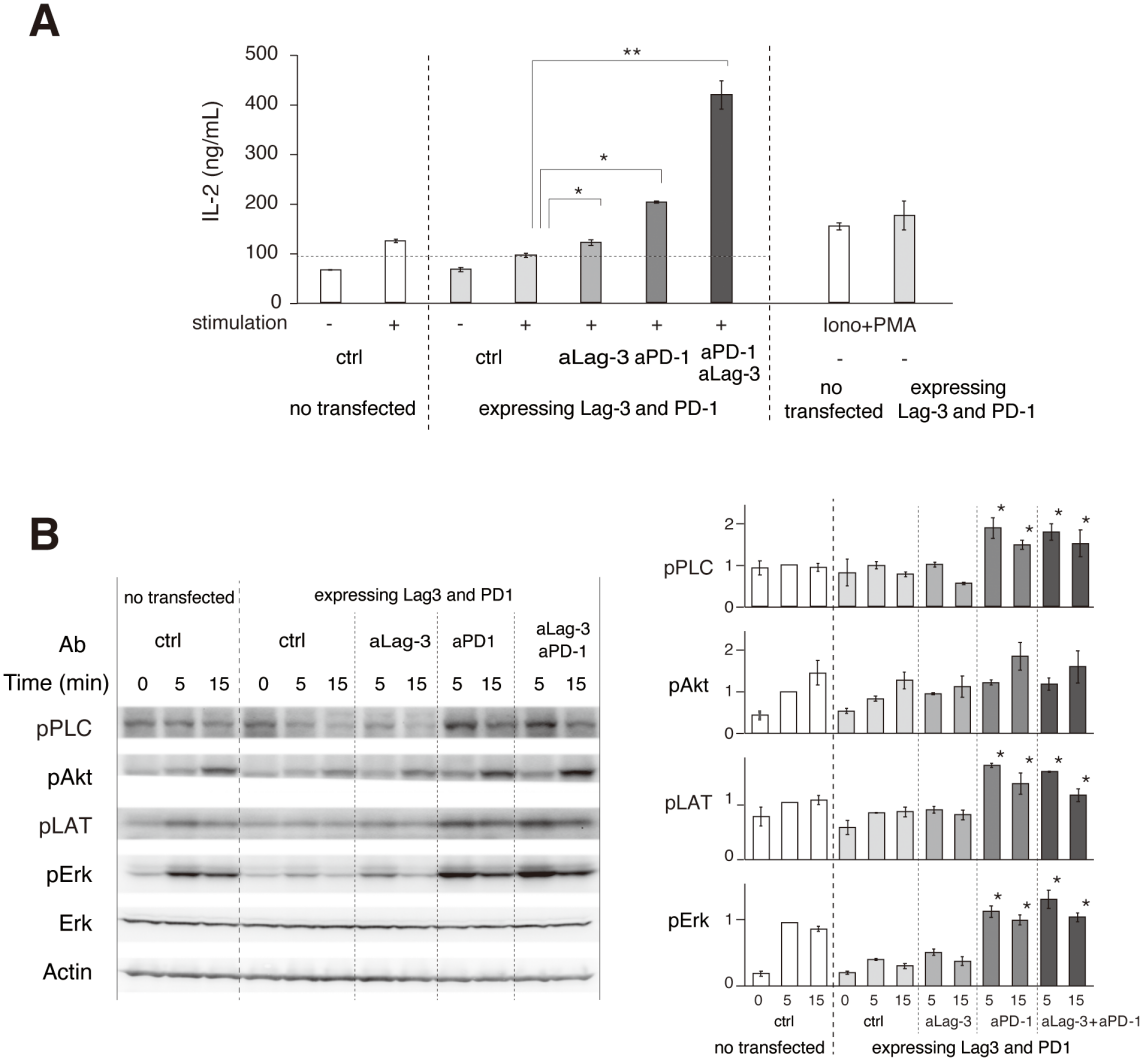
Biochemical effects of aLag-3 and aPD-1 Ab treatment. **(A)** 2D12 T-cell hybridomas expressing Lag-3 and PD-1 were stimulated with MCC peptide and DC-1 cells with or without addition of 20 nM of control Ab (ctrl), aLag-3 (28G10), aPD-1 (DX400), the cocktail of aLag-3 and aPD-1 Abs, or PMA plus ionophore (Iono+PMA). IL-2 production in the culture supernatant after 24 hours was measured. **(B)** 2D12 T-cell hybridoma expressing Lag-3 and PD-1 was stimulated with MCC peptide, and DC-1 cells with or without blocking Abs were lysed at the indicated time after stimulation. Tyrosine phosphorylation levels were analyzed by Western blotting using indicated anti-phospho-tyrosine Abs. The quantitation of the blots is shown on the right. * and ** mean p < 0.1 and 0.01, respectively. MCC, moth cytochrome *c*; Ab, antibody; PMA, phorbol myristate acetate.

Phosphorylation of TCR upstream signaling molecules was evaluated to biochemically analyze the effects of the cocktail of aLag-3 Ab and aPD-1 Ab. 2D12 T-cell hybridoma expressing Lag-3 and PD-1 was stimulated with MCC-loaded DC-1 cells in the presence or absence of blocking Abs. IL-2 production after 48-hour stimulation revealed that the expression of Lag-3 and PD-1 significantly reduced IL-2 production, and the reduction was restored by the treatment with aLag-3 Ab, aPD-1 Ab, or the cocktail of both Ab (**Figure 5A**) (33–36). The initial phosphorylation levels at 5 min or 15 min after stimulation were analyzed by Western blotting (**Figure 5B**). The level of Erk phosphorylation (pErk) was strongly reduced by the expression of Lag-3 and PD-1. The reduced pErk was slightly recovered by aLag-3 Ab and strongly by aPD-1 Ab; however, there was no additive change to the effect of aPD-1 by the addition of aLag-3. The phosphorylation of PLCγ1, Akt, and LAT was not significantly changed by expressing Lag-3 and PD-1. While aLag-3 Ab treatment did not show any modulation, aPD-1 Ab increased the phosphorylation of these upstream signaling molecules. However, again, the addition of aLag-3 Ab to aPD-1 Ab did not induce any additional effect. The discrepant effect between increased IL-2 production at later times by adding aLag-3 to aPD-1 and minimal changes in phosphorylation by adding aLag-3 to aPD-1 could be explained by differential time courses (25–27). Imaging and Western blotting were analyzed within the initial few minutes after TCR activation, whereas cytokine production was analyzed 24 hours later. It is possible that the influence of adding aLag-3 to aPD-1 on biochemical endpoints may be more relevant at a later stage of activation.

### 3.6 Efficacy of bispecific Lag-3/PD-1 modalities on cluster formation and function

**Figure 6:**
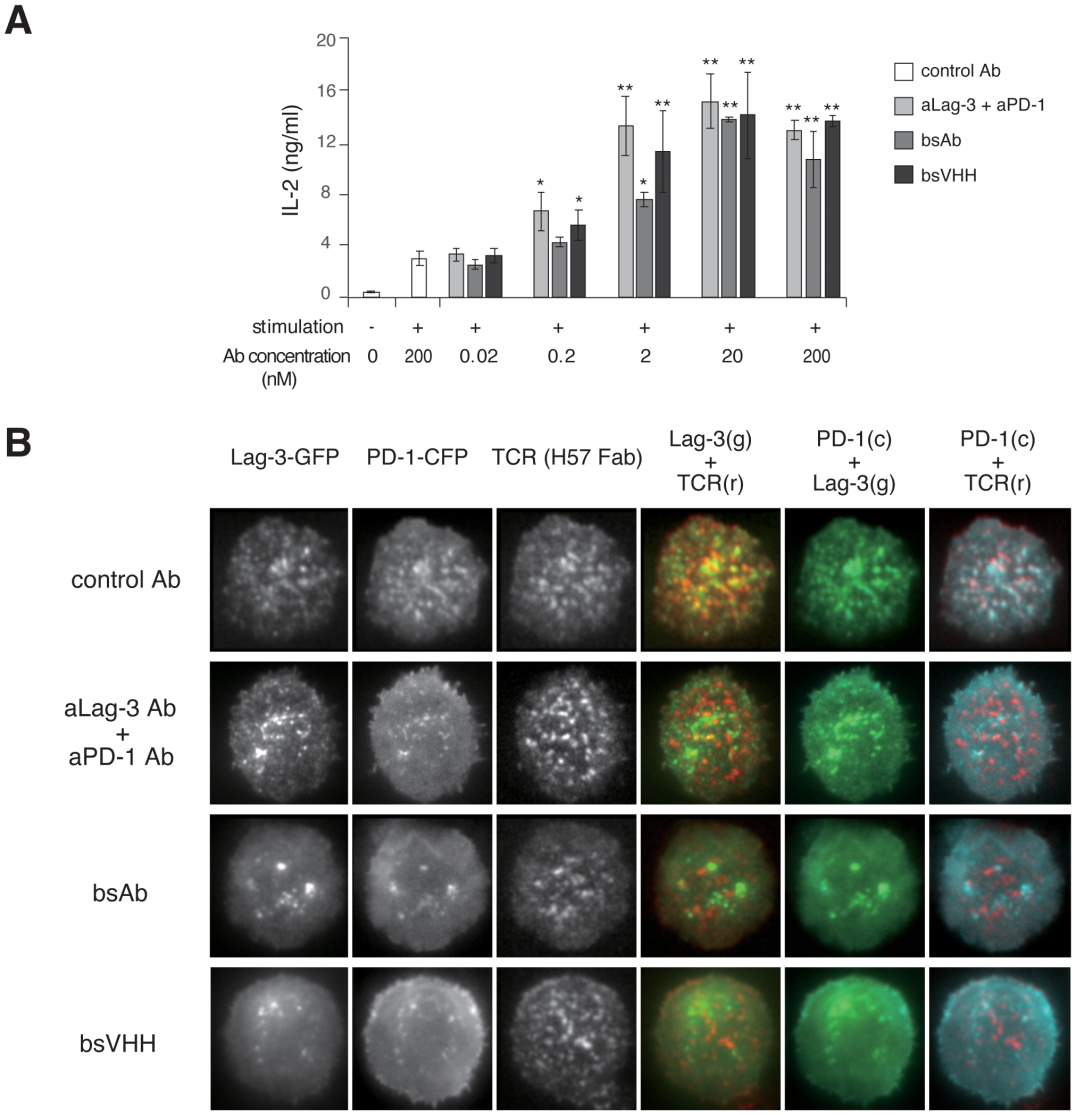
Effects of bispecific Lag-3/PD-1 Abs on Lag-3 and PD-1 cluster formation and function. **(A)** Naïve AND-tg T cells were stimulated with MCC and DC-1 cells with or without control IgG, the cocktail of aLag-3 and aPD-1 Abs, bsAb, or bsVHH. IL-2 production in the culture supernatant after 24 hours was measured. * and ** mean p < 0.1 and 0.01, respectively. **(B)** Representative images of AND-tg T cells co-expressing Lag-3-GFP, PD-1-CFP, and TCR upon Ag stimulation on planar bilayer in the presence of control Ab, the cocktail of aLag-3 and aPD-1 Abs, bsAb, or bsVHH are shown. Abs, antibodies; MCC, moth cytochrome *c*; TCR, T-cell receptor.

We analyzed the efficacy of different bispecific Lag-3/PD-1 construct designs on T-cell function and cluster formation. Dual neutralization of Lag-3 and PD-1 in the T-cell culture was accomplished using three modalities: a cocktail of aLag-3 and aPD-1 Abs, a bispecific aLag-3/PD-1 Ab (bsAb), and a bispecific aLag-3/PD-1 VHH (bsVHH). All of these blocking modalities enhanced IL-2 production, especially at high concentrations (20 nM and 200 nM). The effects of the bsAb were smaller than those of the cocktail or bsVHH at lower concentrations (0.2 nM and 2 nM) (**Figure 6A**). Imaging analysis showed the differential effects of these modalities on Lag-3 cluster formation. The cocktail of aLag-3 and aPD-1 inhibited PD-1 cluster formation, but Lag-3 clusters remained similar to the data in **Figure 4A** (**Figure 6B** for imaging and **Supplementary Figure 5** for quantification). Both bispecific modalities inhibited Lag-3 cluster formation, and the resulting images of Lag-3 were almost the same as those of PD-1, suggesting that Lag-3 and PD-1 may be tightly associated together by bispecific modalities and possibly by PD ligands. The effect of the combination of Abs and bsVHH on IL-2 production was almost the same as that of imaging data.

**Figure 7:**
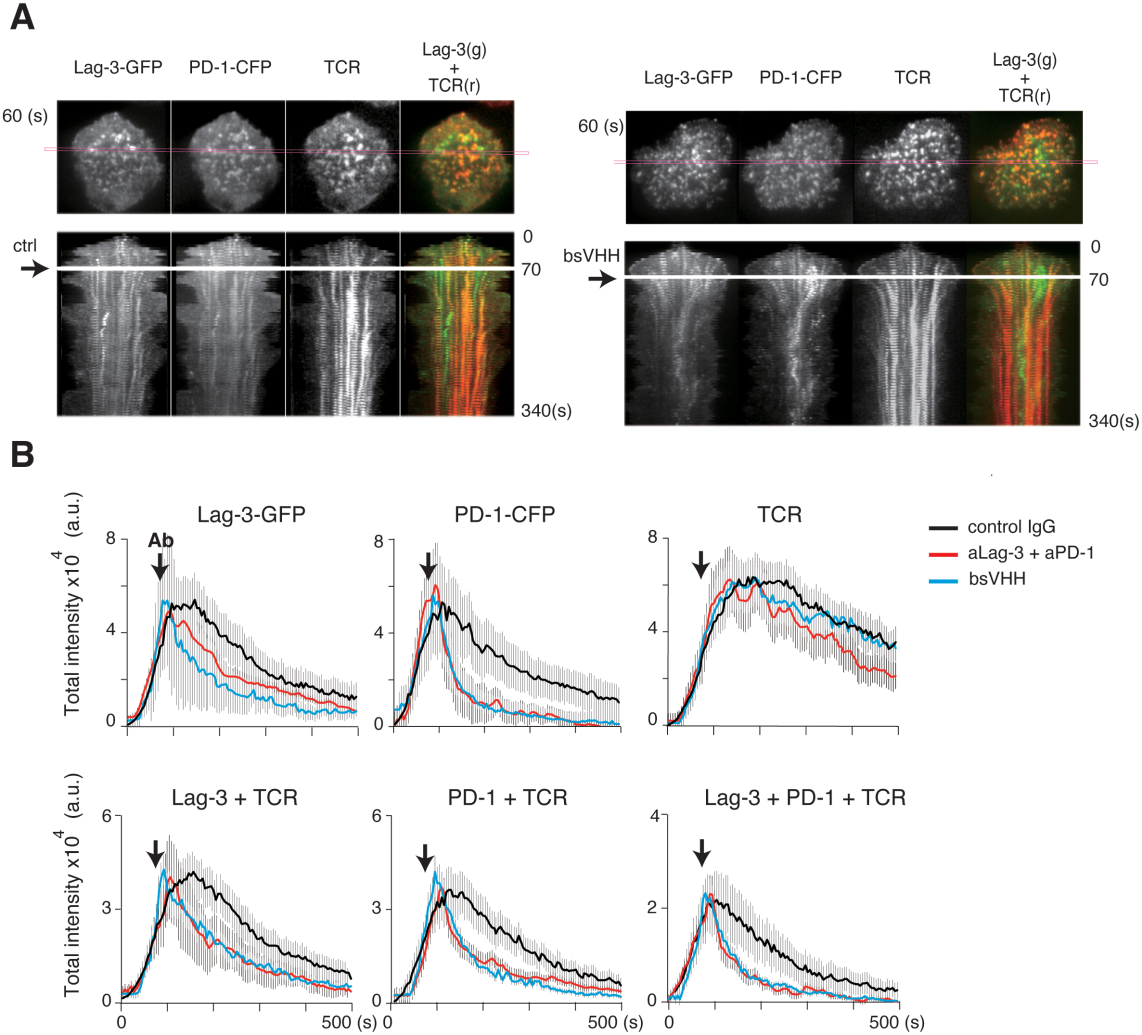
Immediate effects of blocking Abs after starting TCR activation. AND-tg T cells expressing Lag-3-GFP and PD-1-CFP were stimulated with Ag peptide on planar bilayer in the presence of control IgG (ctrl), the cocktail of aLag-3 and aPD-1 Abs, or Lag-3/PD-1 bsVHH, which were added at 60 seconds after initial cluster formation (arrow). **(A)** Representative images of Lag-3-GFP, PD-1-CFP, and TCR around 60 sec after stimulation (upper) and their time-dependent kymographs at the indicated cross-section of the cell (magenta square) after adding control Ab (ctrl) or bsVHH are shown (lower). **(B)** Statistical analysis of **Figure 6A**; the total cluster intensities of Lag-3-GFP, PD-1-CFP, TCR, co-localized clusters of Lag-3 and TCR, PD-1 and TCR, and triple co-localized clusters (Lag-3+PD-1+TCR) are plotted versus time. Abs, antibodies; TCR, T-cell receptor.

Finally, we analyzed whether bispecific modalities could therapeutically block the ongoing activation process even after TCR activation had started to resemble the *in vivo* checkpoint therapy scenario. Control IgG, a cocktail of aLag-3 and aPD-1 Ab, or bsVHH was added 60 sec after initial cluster formation in the planer bilayer system, and images were simultaneously analyzed for the Lag-3, PD-1, and TCR-MC formation (**Figure 7A**). The red lines within the cells (top panels) correspond to the kymograph (lower panels) (**Figure 7A**), and the statistical analysis (**Figure 7B**) is shown. In the kymographs, clusters started to dissolve as soon as the cocktail of Abs or bsVHH was added. Total cluster intensities of Lag-3 and PD-1, the dual co-localizing cluster of Lag-3/TCR and PD-1/TCR, and the triple co-localizing cluster of Lag-3/PD-1/TCR were immediately reduced, whereas TCR-MCs did not change (**Figure 7B**). These data clearly demonstrate that either the cocktail of two Abs or bsVHH inhibits Lag-3 and PD-1 cluster formation and the co-localization between PD-1 or Lag-3 and TCR-MCs during ongoing TCR-MC formation. The separation of the clusters of PD-1 and Lag-3 from TCR-MCs leads to augmentation of T-cell activation.

## 4 Discussion

Lag-3 is expressed by T cells upon Ag stimulation, and thus, all activated effector T cells express Lag-3 and are susceptible to Lag-3-mediated inhibition of activation. Exhausted T cells in chronic viral infection or a tumor microenvironment express high levels of PD-1 and Lag-3, which suppress activation to keep T cells hypo-responsive. aLag-3 Abs are efficacious as a tumor ICI therapy likely by impacting T cell-mediated immunity versus effects on natural killer (NK) cells or monocyte/DC activation (28). We analyzed the mechanism of Lag-3-mediated inhibition of T-cell activation using imaging analysis as well as the effects of ICIs on Lag-3, PD-1, and TCR spatial and temporal distribution.

To mimic the situation in effector T cells *in vivo*, Lag-3-GFP was expressed in AND-tg T cells, and the behavior of Lag-3 was analyzed on a planer bilayer expressing Ag/MHC-II using TIRF microscopy. Lag-3 generated clusters upon T-cell stimulation, which co-localized with TCR-MC; the addition of an MHC-II blocking aLag-3 Ab prevented the co-localization of Lag-3 clusters with TCR-MC. Co-localization between TCR-MC and Lag-3 clusters may reflect the interaction between Lag-3 and TCR-MC, which was also recently demonstrated by Guy et al. (29). Whereas PD-1 clusters disappeared following the addition of aPD-1 Abs, Lag-3 clusters did not disappear and were detected even after the dissociation of Lag-3 clusters from TCR-MC. The behavior of the Lag-3 cluster after Ab treatment is one difference between Lag-3 and PD-1. There is a possibility that bivalent Lag-3 Ab promoted Lag-3 endocytosis and that intracellular Lag-3 aggregates were being detected by TIRF; however, the Lag-3 is likely present on the cell surface since Lag-3 was still detected following monovalent OAA aLag-3 Ab and monovalent aLag-3 Fab treatment.

Maruhashi et al. suggested that Lag-3 has a high affinity to a stable peptide/MHC-II complex, suggesting that the affinity of peptide to MHC-II is critical for Lag-3 binding to MHC-II (30). In contrast, our results showed that Lag-3 cluster formation and co-localization with TCR-MCs depend on the strength of TCR activation (**Figure 3A**). These results are not inconsistent considering the different experimental conditions in which Lag-3 was evaluated. The previous study used a soluble pentameric form of Lag-3’s extracellular domain (Lag-3-EC), whereas we used Lag-3-GFP protein expressed by living T cells with analysis before and during cluster formation. The Lag-3 cluster on the cell surface upon strong stimulation may have a higher affinity to the peptide/MHC-II complex.

It is widely believed that inhibition of Lag-3 binding to MHC-II is critical in neutralizing Lag-3’s inhibitory function. Indeed, 28G10, a potent inhibitor of Lag-3/MHC-II binding, works as ICI to enhance T-cell function *in vitro* and *in vivo*. Most aLag-3 mAbs are similar to 28G10 due to being identified based on screening for the MHC-II blockade attribute. We found, however, that aLag-3 clone 17D8 and C9B7W that demonstrate weak-to-no MHC-II blockade in a biophysical assay still functionally enhanced T-cell activity similar to the potent MHC-II blocking 28G10. These results suggest that the inhibitory function of Lag-3 does not necessarily depend on MHC-II engagement; these results may also be related to data from Maruhashi et al. demonstrating Lag-3 binds to MHC-II only with the strong association of TCR and peptide/MHC-II (30). In addition, evidence that Lag-3 does not necessarily function solely in an MHC-II-dependent fashion was shown in two experimental systems: one was that T-cell activation was enhanced in the presence of aLag-3 Ab when T cells were activated by immobilized CD3 Abs in the absence of MHC-II, and the other was that Lag-3 mediated suppression even in CD8 T cells recognizing peptide/MHC-I complex. The association of Lag-3 with TCR-MC induces inhibition of activation even in the absence of Lag-3/MHC-II association.

The mechanism of how TCR-MC and Lag-3 clusters are assembled is not clear. Since some aLag-3 Abs such as C9B7W, which is not an MHC-II blocking Ab, inhibit the assembly, Lag-3 associates through the extracellular domain. It has been recently shown that Lag-3 forms a dimer and C9B7W blocks the dimer formation, resulting in functional inhibition (31). Therefore, the association between TCR-MC and Lag-3 cluster may also be mediated through Lag-3 dimer, which could be inhibited by these aLag-3 Abs.

The cocktail of aLag-3 and aPD-1 Ab showed enhanced effects on IL-2 production at 48 hours (**Figures 4C**, **5A**). The cocktail, however, showed mere additive effect but no synergistic effect on the co-localized clusters of Lag-3, PD-1, and TCR clusters at 10 min (**Figures 4A, B**) or initial phosphorylation levels at 15 min (**Figure 5B**). These results reveal that the improved effect occurs at least greater than 15 min after TCR activation has started. Such delay may relate to the expression of Lag-3 and PD-1. It is known that the treatment by aPD-1/aPD-L1 Abs upregulates Lag-3 expression (32). aPD-1 Ab treatment alleviating PD-1-mediated inhibition leads to greater T-cell activation, which upregulates Lag-3 expression to a greater extent, leading to stronger Lag-3-dependent inhibition. The inverse situation is expected following aLag-3 treatment. In contrast, the treatment with a cocktail of aLag-3 and aPD-1 Abs leads to greater T-cell activation and upregulation of both Lag-3 and PD-1; however, these Abs block their inhibitory functions. Consequently, the increase of IL-2 produced by the cocktail treatment would become larger than that by the simple summation of aLag-3 and aPD-1 Ab treatment increase.

Treatment with bsAb and bsVHH resulted in the disappearance of Lag-3 clusters, although Lag-3 clusters remained after aLag-3 treatment (**Figure 6B**). Considering the observation that aPD-1 Ab induced the disappearance of PD-1 clusters, it was conceivable that the bispecific modalities force the association of Lag-3 with PD-1 preventing Lag-3 cluster formation. Furthermore, we showed that bsVHH functions similarly to a cocktail of aLag-3 and aPD-1 Ab. Considering that bsVHH may have additional tumor diffusion advantages due to its smaller size compared to larger Ab, bsVHH may also have a differentiated anti-tumor activity *in vivo*.

Our results reveal that effective ICIs should target to dissociate inhibitory receptors such as PD-1 and Lag-3 from the TCR-MC at the immune synapse. This novel mechanism of action should be considered for further development of effective immune therapy.

## Data Availability

The original contributions presented in the study are included in the article/**Supplementary Material**. Further inquiries can be directed to the corresponding author.

## Supplementary material

Supplementary Figure 1 Time course of surface expression of Lag-3 and PD-1. **(A)** Purified naïve AND-tg T cells were stimulated with MCC and irradiated splenocyte. Surface Lag-3, PD-1, Tigit, CD3ε, and CD25 were analyzed by flow cytometry on the indicated days. Lag-3 expression levels gradually increased and kept higher levels from day 3 to 7. Lag-3 up-regulation was more transient compared to the other co-inhibitory molecule PD-1 and Tigit. **(B)** AND-tg T cells stimulated with MCC/APC for 3 days (left), and T cells from Lag-3-KO AND-tg mice were stimulated and Lag-3-GFP was retrovirally transfected and Lag-3+ cells were sorted and analyzed (right). Both T cells were stained with the same aLag-3 Ab.

Supplementary Figure 2 The method to evaluate number and intensity of clusters. **(A)** First, original image stacks were processed by Fiji. The backgrounds were subtracted by rolling ball background subtraction method and the contrast was automatically adjusted around the maximum expansion time and converted to 8 bits from 16 bit. **(B, C)** Next, the processed image stacks were analyzed by IMARIS. The co-localized clusters were extracted and the cluster numbers **(B)** and the intensities **(C)** of GFP, TCR, and co-localized clusters counted. **(B)** The cluster number (n) failed to represent impressions of the images because very small and big clusters were being equally counted. As the result, the difference between 10 μM and 100 nM MCC was very small. **(C)** In contrast to cluster number, the parameter derived from multiplying the cluster number (n) and the cluster intensity (a.u.) were plotted as total cluster intensity (a.u.). This value represents the differences between 10 μM and 100 nM MCC very well.

Supplementary Figure 3 Blocking ability of a panel of aLag-3 Abs on Lag-3/MHC-II association. A20 cells were pre-incubated with recombinant mLAG-3-Fc in the presence of a panel of aLag-3 Abs; control rat IgG (ctrl), Lag-3/MHC-II blocking Ab (28G10), non/marginal-blocking aLAG-3 Abs (C9B7W, 17D8), and non-blocking Abs (1F3, 9B7). Lag-3/MHC-II binding was assessed by detecting the bound mLag3-Fc by flow cytometry.

Supplementary Figure 4 Requirement of MHC-II binding for cluster formation and inhibitory function of Lag-3. **(A)** Various Lag-3 deletion mutants (wild-type Lag-3-GFP; 1^st^ domain-deletion mutant Lag-3(234)-GFP; 2^nd^, 3^rd^ and 4^th^ domain-deletion mutant Lag-3(1)-GFP; and chimeric mutant with 1^st^ domain of Lag-3 and three domains of human CD22 as Lag-3(1)hCD22(345)) were expressed in 2D12 T hybridomas and activated T cells were analyzed on planer bilayer. All the mutants formed Lag-3 clusters but failed to co-localize with TCR-MCs. The peak values of total intensities were plotted on top right. **(B)** Wild-type and mutant Lag-3 expressing 2D12 T cell hybridomas which were stimulated with 10 μM MCC peptide and DC-1 cell line (top) or plate-bound aCD3ε/aCD28 Ab (bottom) and IL-2 produced in the culture supernatant after 48 hours was measured. Wild-type Lag-3 expression inhibited IL-2 production while all the other Lag-3 mutants had no effect. **(C)** The effects of aLag-3 Ab on CD8 T cells. Purified naïve OT-1 T cells (top) or effector T cells (bottom) were stimulated with ova peptide presented by irradiated splenocytes from wild-type (WT) or MHC-II KO mice in the presence of control IgG (ctrl), aLag-3 Ab, aPD-1 Ab or the cocktail of aLag-3 and aPD-1 Abs (aLag-3 + aPD-1). IL-2 production after 48 hours was measured. (Horizontal dotted lines were shown just for comparison). While the effect of aLag-3 in naïve T-cells was smaller using MHC-II KO APC, the enhancing effect by aLag-3 in effector T cells was totally absent using MHC-II KO APC. **(D)** The effects of aLag-3 Ab on T cells upon MHC-II-independent stimulation. Parental (not transfected) and dual Lag-3+PD-1-transfected 2D12 T cell hybridomas were stimulated with plate-bound anti-CD3/CD28 Abs in the presence of control IgG (ctrl), aLag-3, aPD-1 or the cocktail of aLag-3 and aPD-1 Abs (aLag-3 + aPD-1) and IL-2 produced in the supernatant measured. Lag-3 and PD-1 expression inhibited IL-2 production. (Horizontal dotted lines were shown just for comparison). Whereas aPD-1 had no effect, aLag-3 increased IL-2 production in the absence of MHC-II suggesting that Lag-3 has an MHC-II-independent inhibitory function. * and ** means p<0.1 and 0.01, respectively.

Supplementary Figure 5 Effects of bispecific modalities on Lag-3 and PD-1 clusters. The peak values of total cluster intensities for the images shown in **Figure 6B** were plotted. The results from the images of Lag-3-GFP, PD-1-CFP, TCR, co-localization of Lag-3 and TCR (Lag-3+TCR), co-localization of PD-1 and TCR (PD-1+TCR), and triple co-localization (PD-1+Lag-3+TCR) by treatment with control IgG (ctrl), the cocktail of aLag-3 Ab and aPD-1 Ab (aLag-3+aPD-1), or bsVHH are shown. * and ** means p< 0.1 and 0.01, respectively.

Supplementary Figure 6 Generation of Lag3-KO mice. Top: mouse genomic locus of the Lag-3 gene. Lag-3 coding exons 1–8 and translational start and stop codons are depicted and the targeted exon 3 highlighted in red. Middle: sequences of the proximal and distal sgRNAs. Bottom: constitutive KO allele after CRISPR/Cas9 mediated gene editing, resulting in deletion of exon 3 and a frameshift in exon 4.

## References

[1] Hegde PS Chen DS. Top 10 challenges in cancer immunotherapy. Immunity. (2020) 52:17–35. doi: 10.1016/j.immuni.2019.12.011 31940268

[2] Barber DL Wherry EJ Masopust D Zhu B Allison JP Sharpe AH. Restoring function in exhausted CD8 T cells during chronic viral infection. Nature. (2006) 439:682–7. doi: 10.1038/nature04444 16382236

[3] Held W Siddiqui I Schaeuble K Speiser DE. Intra-tumoral CD8^+^ T cells with stem-like properties: Implications for cancer immunotherapy. Sci Transl Med. (2019) 11:eaay6863. doi: 10.1126/scitranslmed.aay6863 31645454

[4] Marrone KA Ying W Naidoo J. Immune-related adverse events from immune checkpoint inhibitors. Clin Pharmacol Ther. (2016) 100:242–51. doi: 10.1002/cpt.394 27170616

[5] Pitt JM Vétizou M Daillère R Roberti MP Yamazaki T Routy B. Resistance mechanisms to immune-checkpoint blockade in cancer: tumor-intrinsic and -extrinsic factors. Immunity. (2016) 44:1255–69. doi: 10.1016/j.immuni.2016.06.001 27332730

[6] Gellrich FF Schmitz M Beissert S Meier F. Anti-PD-1 and novel combinations in the treatment of melanoma-an update. J Clin Med. (2020) 9:223. doi: 10.3390/jcm9010223 31947592 PMC7019511

[7] Cheng AL Hsu C Chan SL Choo SP Kudo M. Challenges of combination therapy with immune checkpoint inhibitors for hepatocellular carcinoma. J Hepatol. (2020) 72:307–19. doi: 10.1016/j.jhep.2019.09.025 31954494

[8] De Sousa Linhares A Leitner J Grabmeier-Pfistershammer K Steinberger P. Not all immune checkpoints are created equal. Front Immunol. (2018) 9:1909. doi: 10.3389/fimmu.2018.01909 30233564 PMC6127213

[9] Triebel F Jitsukawa S Baixeras E Roman-Roman S Genevee C Viegas-Pequignot E. LAG-3, a novel lymphocyte activation gene closely related to CD4. J Exp Med. (1990) 171:1393–405. doi: 10.1084/jem.171.5.1393 PMC2187904 1692078

[10] Huard B Prigent P Tournier M Bruniquel D Triebel F. CD4/major histocompatibility complex class II interaction analyzed with CD4- and lymphocyte activation gene-3 (LAG-3)-Ig fusion proteins. Eur J Immunol. (1995) 25:2718–21. doi: 10.1002/eji.1830250949 7589152

[11] Wang J Sanmamed MF Datar I Su TT Ji L Sun J. Fibrinogen-like protein 1 is a major immune inhibitory ligand of LAG-3. Cell. (2019) 176:334–47. doi: 10.1016/j.cell.2018.11.010 PMC6365968 30580966

[12] Woo SR Turnis ME Goldberg MV Bankoti J Selby M Nirschl CJ. Immune inhibitory molecules LAG-3, and PD-1 synergistically regulate T-cell function to promote tumoral immune escape. Cancer Res. (2012) 72:917–27. doi: 10.1158/0008-5472.CAN-11-1620 PMC3288154 22186141

[13] Tawbi HA SChadendorf D Lipson EJ Ascierto PA Matamala L Gutierrez EC. Relatlimab and Nivolumab versus Nivolumab in untreated advanced melanoma. N Engl J Med. (2022) 386:24–34. doi: 10.1056/NEJMoa2109970 34986285 PMC9844513

[14] Luke JJ Patel MR Blumenschein GR Hamilton E Chmielowski B Ulahannan SV. The PD-1 and LAG-3-targeting bispecific molecule tebotelimab in solid tumors and hematologic cancers; a phage 1 trial. Nat Med. (2023) 29:2814–24. doi: 10.1038/s41591-023-02593-0 PMC10667103 37857711

[15] Wei J Yang Y Wang G Liu M. Current landscape and future directions of bispecific antibodies in cancer immunotherapy. Front Immunol. (2022) 13:1035276. doi: 10.3389/fimmu.2022.1035276 36389699 PMC9650279

[16] Yokosuka T Sakata-Sogawa K Kobayashi W Hiroshima M Hashimoto-Tane A Tokunaga M. Newly generated T cell receptor microclusters initiate and sustain T cell activation by recruitment of Zap70 and SLP-76. Nat Immunol. (2005) 6:1253–62. doi: 10.1038/ni1272 16273097

[17] Yokosuka T Takamatsu M Kobayashi-Imanishi W Hashimoto-Tane A Azuma M Saito T. Programmed cell death 1 forms negative costimulatory microclusters that directly inhibit T cell receptor signaling by recruiting phosphatase SHP2. J Exp Med. (2012) 209:1201–17. doi: 10.1084/jem.20112741 PMC3371732 22641383

[18] Hashimoto-Tane A Yokosuka T Saito T. Analyzing the dynamics of signaling microclusters. Methods Mol Biol. (2017) 1584:51–64. doi: 10.1007/978-1-4939-6881-7_4 28255695

[19] Workman CJ Dugger KJ Vignali DA. Molecular analysis of the negative regulatory function of lymphocyte activation gene-3. J Immunol. (2002) 169:5392–5. doi: 10.4049/jimmunol.169.10.5392 12421911

[20] Cemerski S Zhao S Chenard M Laskey J Cui L Shukla R. T cell activation and anti-tumor efficacy of anti-LAG-3 antibodies is independent of LAG-3-MHCII blocking capacity. J Immunother Cancer. (2015) 3:183. doi: 10.1186/2051-1426-3-S2-P183

[21] Huard B Mastrangeli R Prigent P Bruniquel D Donini S El-Tayar N. Characterization of the major histocompatibility complex class II binding site on LAG-3 protein. Proc Natl Acad Sci USA. (1997) 94:5744–9. doi: 10.1073/pnas.94.11.5744 PMC20850 9159144

[22] Grosso JF Kelleher CC Harris TJ Maris CH Hipkiss EL De Marzo A. LAG-3 regulates CD8+ T cell accumulation and effector function in murine self- and tumor-tolerance systems. J Clin Invest. (2007) 117:3383–92. doi: 10.1172/JCI31184 PMC2000807 17932562

[23] Woo SR Turnis ME Goldberg MV Bunkoti J Selby M Nirschl CJ. Immune inhibitory molecules LAG-3, and PD-1 synergistically regulate T cell function to promote tumor immune escape. Cancer Res. (2012) 72:917–27. doi: 10.1158/0008-5472.CAN-11-1620 PMC3288154 22186141

[24] Matsuzaki J Gnjatic S Mhawech-Fauceglia P Beck A Miller A Tsuji T. Tumor-infiltrating NY-ESO-1-specific CD8+ T cells are negatively regulated by LAG-3 and PD-1 in human ovarian cancer. Proc Natl Acad Sci USA. (2010) 107:7875–80. doi: 10.1073/pnas.1003345107 PMC2867907 20385810

[25] Hannier S Tournier M Bismuth G Triebel F. CD3/TCR complex-associated lymphocyte activation gene-3 molecules inhibit CD3/TCR signaling. J Immunol. (1998) 161:4058–65. doi: 10.4049/jimmunol.161.8.4058 9780176

[26] Bhagwat B Cherwinski H Sathe M Seghezzi W McClanahan K de Waal Malefyt R. Establishment of engineered cell-based assays mediating LAG3 and PD1 immune suppression enables potency measurement of blocking antibodies and assessment of signal transduction. J Immunol Methods. (2018) 456:7–14. doi: 10.1016/j.jim.2018.02.003 29427592

[27] Wang L Yu C Wang K Wang J. A reporter gene assay for measuring the bioactivity of anti-LAG-3 therapeutic antibodies. Luminescence. (2020) 35:1408–15. doi: 10.1002/bio.3905 32598535

[28] Andrews LP Yano H Vignali DAA. Inhibitory receptors and ligands beyond PD-1, PD-L1 and CTLA-4: breakthroughs or backups. Nat Immunol. (2019) 20:1425–34. doi: 10.1038/s41590-019-0512-0 31611702

[29] Guy C Mitrea DM Chou P-C Temirov J Vignali KM Liu S. LAG3 associates with TCR-CD3 complexes and suppresses signaling by driving co-receptor-Lck dissociation. Nat Immunol. (2022) 23:757–67. doi: 10.1038/s41590-022-01176-4 PMC9106921 35437325

[30] Maruhashi T Okazaki IM Sugiura D Takahashi S Maeda TK Shimizu K. LAG-3 inhibits the activation of CD4^+^ T cells that recognize stable pMHCII through its conformation-dependent recognition of pMHCII. Nat Immunol. (2018) 19:1415–26. doi: 10.1038/s41590-018-0217-9 30349037

[31] Silberstein JL Du J Chan K-W Frank JA Mathews II Kim YB. Structural insights reveal interplay between LAG-3 homodimerization, ligand binding, and function. Proc Natl Acad Sci USA. (2024) 121:e2310866121. doi: 10.1073/pnas.2310866121 38483996 PMC10962948

[32] He Y Yu H Rozeboom L Rivard CJ Ellison K Dziadziuszko R. LAG-3 protein expression in non-small cell lung cancer and its relationship with PD-1/PD-L1 and tumor-infiltrating lymphocytes. J Thorac Oncol. (2017) 12:814–23. doi: 10.1016/j.jtho.2017.01.019 28132868

[33] Jovčevska I Muyldermans S. The therapeutic potential of nanobodies. Bio Drugs. (2020) 34:11–26. doi: 10.1007/s40259-019-00392-z PMC6985073 31686399

[34] Runcie K Budman DR John V Seetharamu N. Bi-specific and tri-specific antibodies- the next big thing in solid tumor therapeutics. Mol Me. (2018) 24:50. doi: 10.1186/s10020-018-0051-4 PMC6154901 30249178

[35] Gong J Chehrazi-Raffle A Reddi S Salgia R. Development of PD-1 and PD-L1 inhibitors as a form of cancer immunotherapy: a comprehensive review of registration trials and future considerations. J Immunother Cancer. (2018) 6:8. doi: 10.1186/s40425-018-0316-z 29357948 PMC5778665

[36] Chocarro L Blanco E Arasanz H Fernandez-Rubil L Bocanegra Echaide M. Clinical landscape of LAG-3-targeted therapy. Immuno-Oncol Technol. (2022) 14:1–9. doi: 10.1016/j.iotech.2022.100079 PMC9216443 35755891

