# Supplementary material for: Dissociation of LAG-3 inhibitory cluster from TCR microcluster by immune checkpoint blockade
Source: Front Immunol. 2024 Aug 21;15:1444424. doi: 10.3389/fimmu.2024.1444424 (PMC11371725; doi:10.3389/fimmu.2024.1444424)

Supplementary Figure 1

A

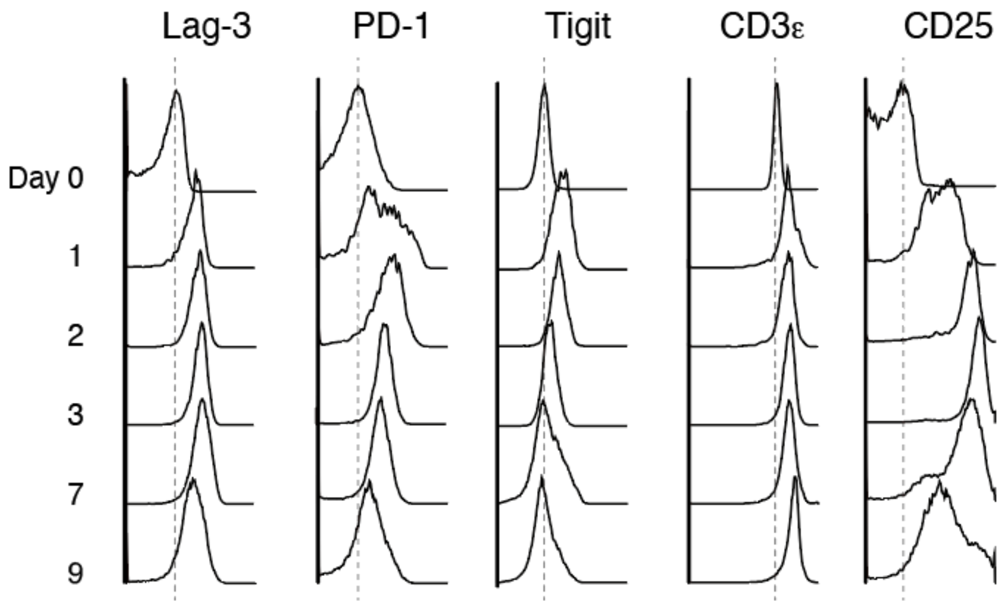

B

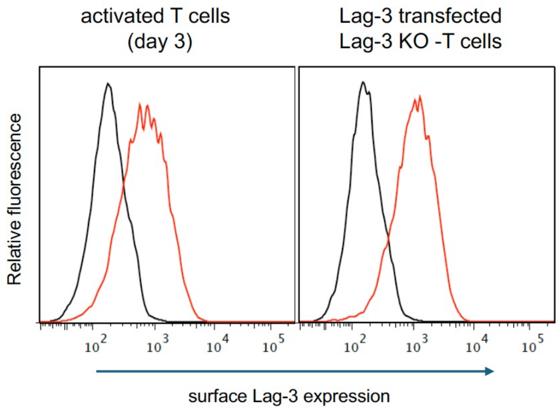

Supplementary Figure 2

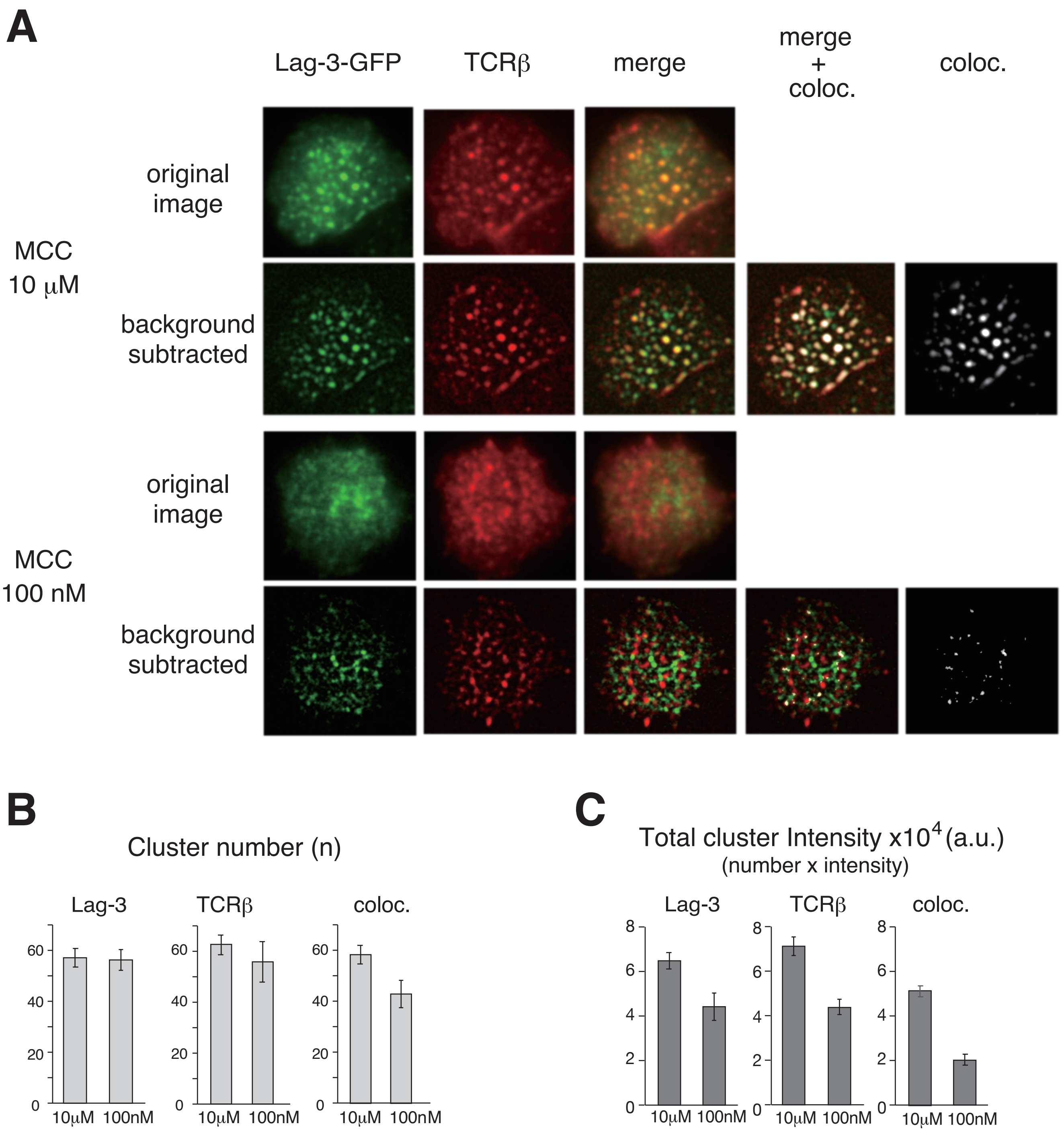

Supplementary Figure 3

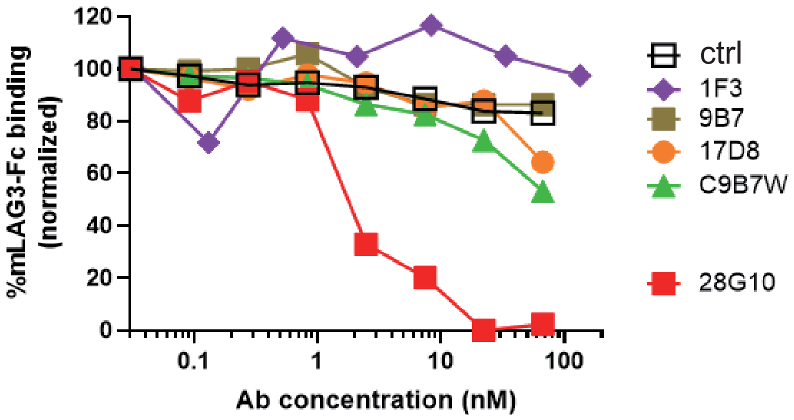

Supplementary Figure 4

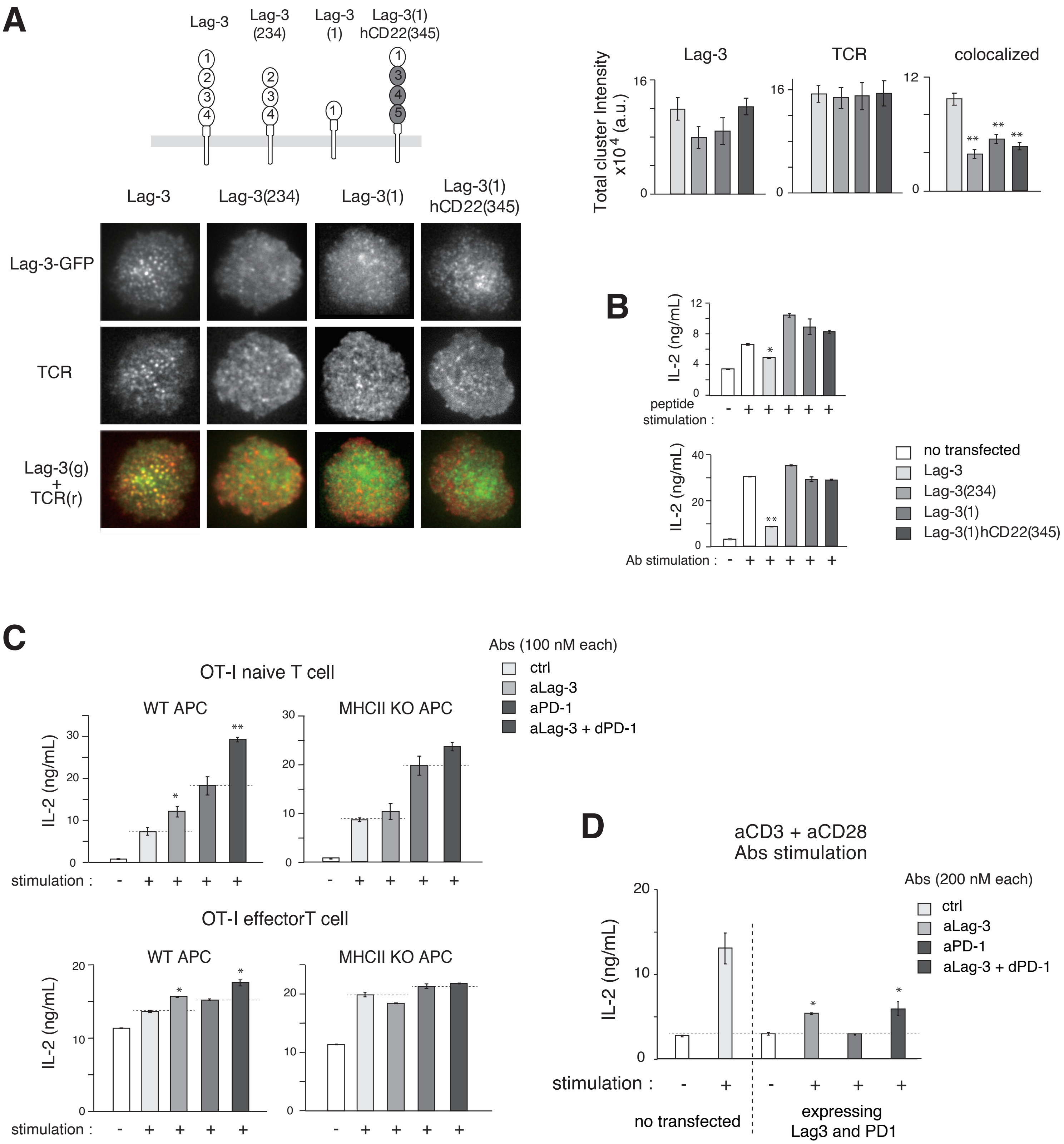

Supplementary Figure 5

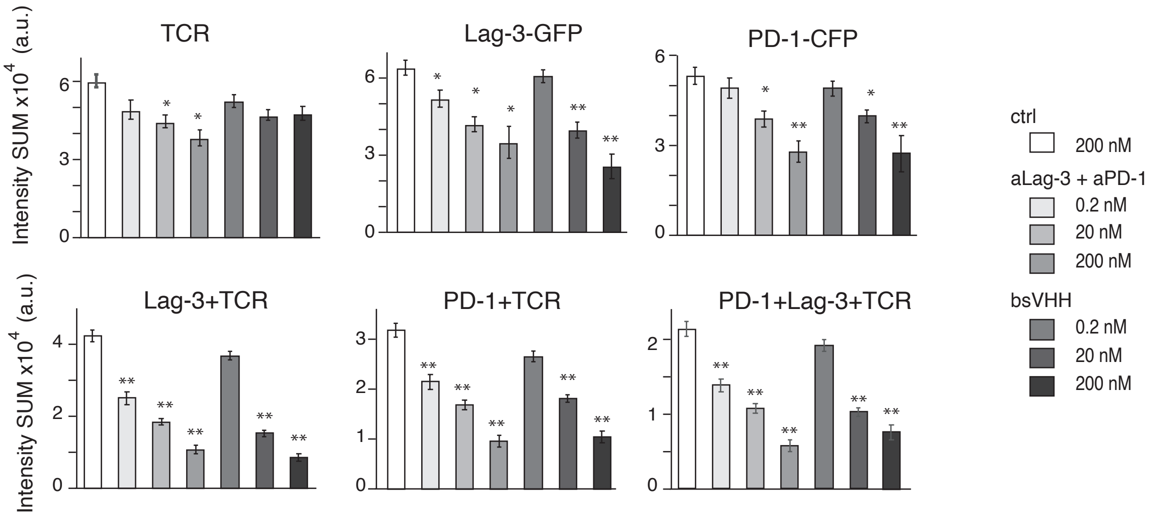

Supplementary Figure 6

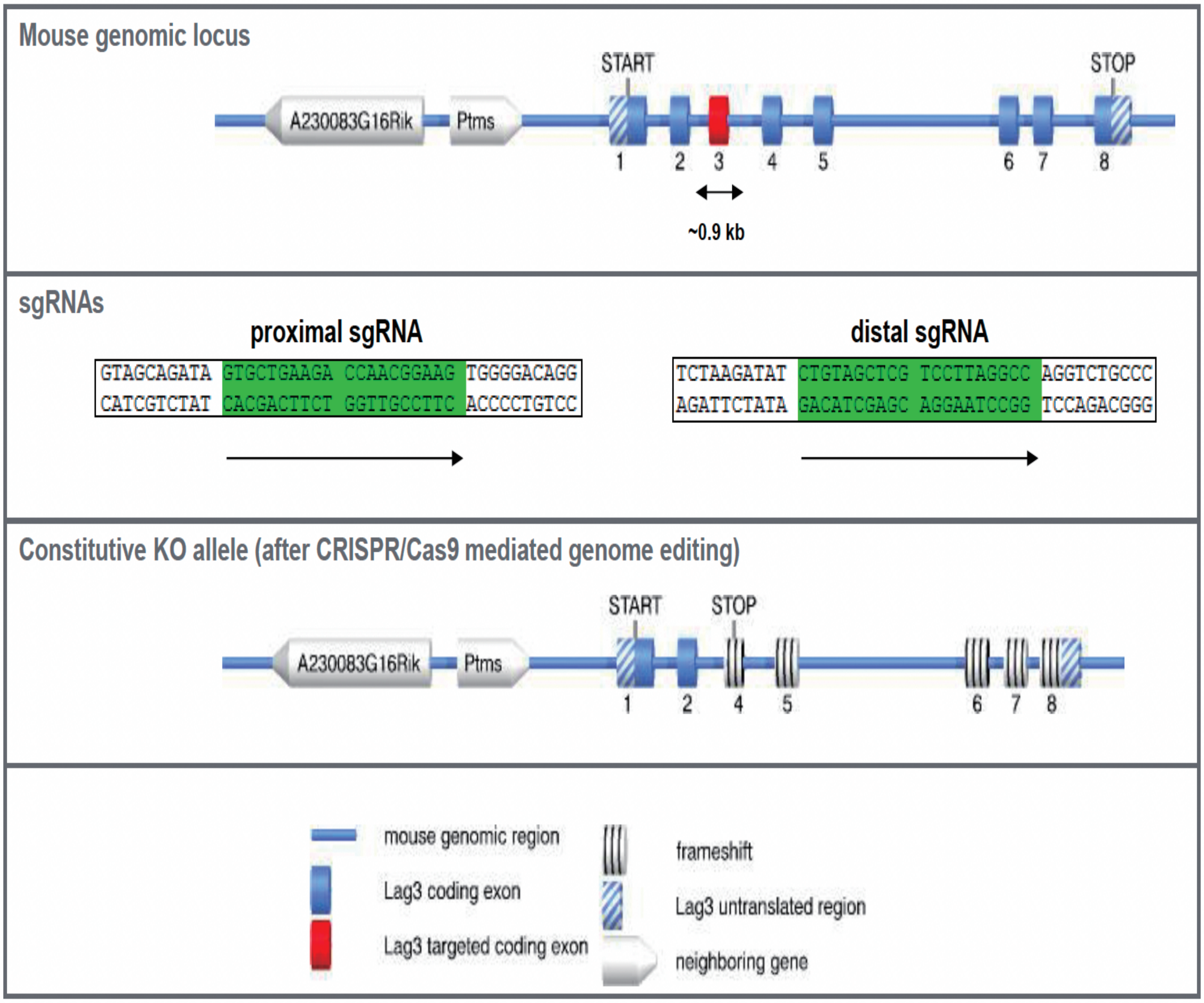

Supplement: Supplementary Figure 1 — Time course of surface expression of Lag-3 and PD-1. (A) Purified naïve AND-tg T cells were stimulated with MCC and irradiated splenocyte. Surface Lag-3, PD-1, Tigit, CD3ε, and CD25 were analyzed by flow cytometry on the indicated days. Lag-3 expression levels gradually increased and kept higher levels from day 3 to 7. Lag-3 up-regulation was more transient compared to the other co-inhibitory molecule PD-1 and Tigit. (B) AND-tg T cells stimulated with MCC/APC for 3 days (left), and T cells from Lag-3-KO AND-tg mice were stimulated and Lag-3-GFP was retrovirally transfected and Lag-3+ cells were sorted and analyzed (right). Both T cells were stained with the same aLag-3 Ab. [file DataSheet1.pdf]
